# Temperature-dependent trophic associations modulate soil bacterial communities along latitudinal gradients

**DOI:** 10.1093/ismejo/wrae145

**Published:** 2024-08-08

**Authors:** Xing Huang, Jianjun Wang, Kenneth Dumack, Karthik Anantharaman, Bin Ma, Yan He, Weiping Liu, Hongjie Di, Yong Li, Jianming Xu

**Affiliations:** Zhejiang Provincial Key Laboratory of Agricultural Resources and Environment, College of Environmental and Resource Sciences, Zhejiang University, Hangzhou 310058, China; State Key Laboratory of Lake Science and Environment, Nanjing Institute of Geography and Limnology, Chinese Academy of Sciences, Nanjing 210008, China; Institute of Zoology, Terrestrial Ecology, Cluster of Excellence on Plant Sciences (CEPLAS), University of Cologne, Cologne 50674, Germany; Department of Bacteriology, University of Wisconsin-Madison, Madison, WI 53705, United States; Zhejiang Provincial Key Laboratory of Agricultural Resources and Environment, College of Environmental and Resource Sciences, Zhejiang University, Hangzhou 310058, China; Zhejiang Provincial Key Laboratory of Agricultural Resources and Environment, College of Environmental and Resource Sciences, Zhejiang University, Hangzhou 310058, China; MOE Key Laboratory of Environmental Remediation and Ecosystem Health, College of Environmental and Resource Sciences, Zhejiang University, Hangzhou 310058, China; Zhejiang Provincial Key Laboratory of Agricultural Resources and Environment, College of Environmental and Resource Sciences, Zhejiang University, Hangzhou 310058, China; Zhejiang Provincial Key Laboratory of Agricultural Resources and Environment, College of Environmental and Resource Sciences, Zhejiang University, Hangzhou 310058, China; Zhejiang Provincial Key Laboratory of Agricultural Resources and Environment, College of Environmental and Resource Sciences, Zhejiang University, Hangzhou 310058, China

**Keywords:** latitudinal gradient, protists, T4-like virus, bacteria, putative trophic interaction, assembly process

## Abstract

Understanding the environmental and biological mechanisms shaping latitudinal patterns in microbial diversity is challenging in the field of ecology. Although multiple hypotheses have been proposed to explain these patterns, a consensus has rarely been reached. Here, we conducted a large-scale field survey and microcosm experiments to investigate how environmental heterogeneity and putative trophic interactions (exerted by protist–bacteria associations and T4-like virus–bacteria associations) affect soil bacterial communities along a latitudinal gradient. We found that the microbial latitudinal diversity was kingdom dependent, showing decreasing, clumped, and increasing trends in bacteria, protists, and T4-like viruses, respectively. Climatic and edaphic drivers played predominant roles in structuring the bacterial communities; the intensity of the climatic effect increased sharply from 30°N to 32°N, whereas the intensity of the edaphic effect remained stable. Biotic associations were also essential in shaping the bacterial communities, with protist–bacteria associations showing a quadratic distribution, whereas virus–bacteria associations were significant only at high latitudes. The microcosm experiments further revealed that the temperature component, which is affiliated with climate conditions, is the primary regulator of trophic associations along the latitudinal gradient. Overall, our study highlights a previously underestimated mechanism of how the putative biotic interactions influence bacterial communities and their response to environmental gradients.

## Introduction

Understanding how abiotic and biotic factors influence species distribution and community structure is critical for the exploration of biogeography [1–3]. As the importance of soil microorganisms, especially soil bacteria, in biogeochemical cycles, crop productivity, and human health is increasingly recognized [4, 5], a growing body of research is emphasizing the elucidation of full microbial diversity, including community structure, assembly, and ecological drivers. Although many studies have shown that multiple abiotic factors control bacterial diversity [6, 7], we lack studies explicitly incorporating environmental factors with characteristics of biotic interactions to determine the relative contributions of abiotic and biotic factors shaping microbial community diversity.

The rice paddy ecosystem is one of the Earth’s largest wetlands and harbors diverse microorganisms that interact strongly with each other due to the aerobic and anaerobic cycles caused by artificial flooding [8]. Among them, protistan predation and viral lysis, as two important top–down control elements, alter the growth, metabolism, and evolutionary strategies of bacterial cells in soil environments [9–11], thereby influencing the coexistence of bacterial species. Recently, it has been shown that the simultaneous presence of protists and viruses strongly affects bacterial virulence and diversification [12, 13]. However, empirical studies have mainly focused on the impacts of soil protists and viruses on bacterial communities based on laboratory incubation experiments or simple model systems [14]. Understanding the latitudinal patterns and driving mechanisms of trophic associations remains difficult at continental scales.

The classical latitudinal biotic interaction hypothesis (LBIH) posits that biotic interactions are more intense in stable and warm climates and generally decrease in intensity from low to high latitudes [15, 16]. This theoretical concept partially explains the latitudinal diversity gradient of species, which suggests that flourishing biotic interactions at low latitudes promote coevolution and might result in high species richness [17–19]. Nonetheless, contradicting the LBIH, recent studies have found weak, absent, or even reversed latitudinal patterns in biotic interactions [20–22]. Consequently, it remains unclear whether biotic interactions are stronger at low latitudes, resulting in higher species richness and how biotic interactions respond to environmental changes. Recent studies have revealed that the variation in ecological networks along environmental gradients may reflect the coexistence mechanisms underlying community assembly [23, 24]. The different impacts of abiotic and biotic filtering processes through multiple reinforcing and conflicting effects can manifest in alternating patterns of network structure [25, 26]. Turnover in species composition is an important source of variation in networks along environmental gradients, as interactions are primarily conditional on species co-occurrence. Therefore, investigations of how networks vary temporally or spatially have the potential to provide new insight into how species interactions vary.

Here, we elucidated how putative trophic interactions influence bacterial communities via a cross-latitude field survey and a laboratory microcosm experiment. We characterized the community compositions of bacteria, protists, and T4-like viruses in paddy field soils along a latitudinal gradient ranging from 19°N to 45°N. Next, we depicted two types of biotic associations, namely, protist–bacteria (P–B) associations and virus–bacteria (V–B) associations at the community and species levels, and explored the impacts of putative top–down controls on the bacterial community. We further assessed the latitudinal gradients of biotic associations and elucidated the underlying environmental drivers. Finally, empirical evidence was obtained for the observed temperature-modulated latitudinal distribution of putative trophic interactions by microcosm experiments with artificial manipulations of temperature and soil water content gradients.

## Materials and Methods

### Soil sampling

**Figure 1:**
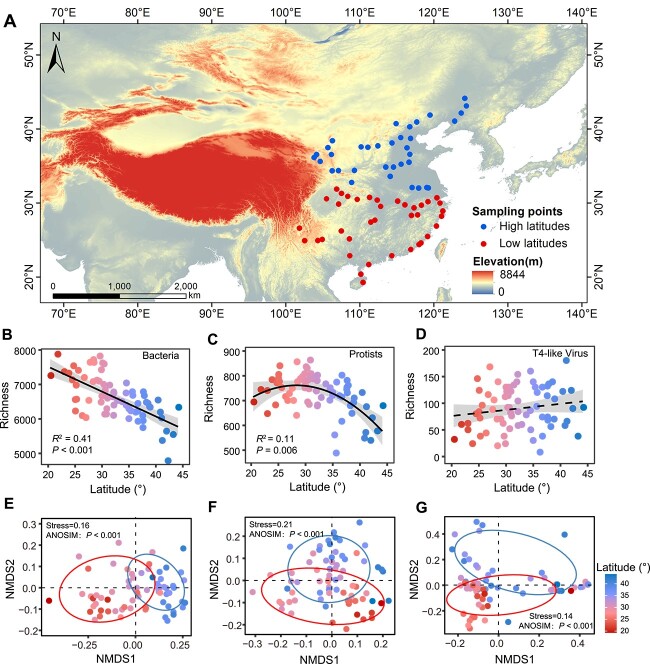
The diversity and structure of microbes in paddy soil along a latitudinal gradient. (a) Geographic distribution of sampled soils. The region from which the soil samples were collected is highlighted with circles. All sampled sites (*n* = 73) are marked and divided into a low latitude group (latitude <32°N) and a high latitude group (latitude >32°N). This separation corresponds to the Qinling Mountains-Huaihe River line (latitude ≈ 32°N), an important geographical (climate, landform, and soil) boundary in Chinese north–south regions. (b–d) Latitudinal distribution of soil microbial alpha-diversity, plotting ASV richness against the latitude of sampling locations. A linear or quadratic model was selected based on the lower value of Akaike’s information criterion. Gray shading indicates the 95% prediction interval. The dotted line represents the nonsignificant (*P* > .05) relationship between latitude and richness. (e–g) NMDS plots based on Bray–Curtis distance show the general patterns of microbial beta-diversity along the latitude. The color gradient denotes the variation in latitude.

During April and May 2016, we collected soil samples from a 100 × 100 m^2^ plot in a paddy field at 76 sites from 28 provinces across Eastern China (Fig. 1a) (19.27°N-47.41°N, 85.12°E-124.41°E). These samples represented a wide range of environmental gradients (such as the climates associated with latitude). The geographical information of the sampling sites is provided in Table S1. Samples were collected from the top 20 cm of paddy soils, and five discrete cores per plot were collected and mixed thoroughly with three replicates. The collected samples were sealed in sterile bags, kept in an icebox, and transported to the laboratory. After field sampling, soils were sieved (<2 mm) and separated into two parts: one was air-dried for physicochemical measurements, and the other was stored at −80°C for DNA extraction.

### Abiotic factor data collection

The geographic distance between pairwise sites was calculated according to the Global Position System (GPS) coordinates of each site. A total of 19 climatic attributes of each sampling site were obtained from the WorldClim database (www.worldclim.org) using the R package “*raster*.” Soil properties were determined according to standardized protocols [27], soil pH was measured in a 1:2.5 ratio of soil to water with a pH meter, soil water content (SWC) was determined after oven-drying at 105°C for 12 h, soil organic matter (SOM) was determined by the dichromate digestion colorimetric method, available phosphorus (AP) was extracted by 0.5 M NaHCO_3_ and determined with the molybdenum blue method, total nitrogen (TN) and total carbon (TC) were determined by dry combustion in a Vario Max CNS analyzer (Elementar Instruments, Mt. Laurel, NJ), nitrate-nitrogen (NO_3_^-^-N), and ammonium-nitrogen (NH_4_^+^-N) was extracted with 1 M KCl and measured using a flow injection analyzer (SAN++, Skalar, Holland) (Table S1). All measurements were measured in triplicate.

To explore the differences in the diversity and composition of microbial communities across different latitudinal regions, we divided the sample sites into two latitudinal groups according to a previous study: a high-latitude group (latitude >32°, *n* = 35) and a low-latitude group (latitude <32°, *n* = 38) [28]. This separation corresponds to the Qinling Mountains-Huaihe River Line (latitude ≈ 32°), an important geographical boundary in Chinese north–south regions regarding climate, landform, and soil conditions [29]. All analyses were performed using R 4.0.2 (http://www.r-project.org), unless otherwise indicated.

### DNA extraction, sequencing, and microbial community characterization

Total microbial genomic DNA was extracted from 0.5 g soil using the FastDNA SPIN Kit for Soil (MP Biomedicals, LLC, Solon). Three replicates of DNA samples were pooled for amplicon sequencing. Three genes targeting distinct taxonomic groups with different taxonomic resolutions were amplified and sequenced using HiSeq System (Illumina): (i) V4 region of the 16S ribosomal RNA (rRNA) genes for bacteria [30], (ii) V9 region of the 18S ribosomal RNA (rRNA) genes for protists [31], and (iii) a fragment of the major capsid protein-encoding gene *g23* of T4-like virus (Table S2) [32].

The Polymerase Chain Reaction (PCR) procedures of 16S rRNA, 18S rRNA, and *g23* were performed in a 20-μl reaction system, which contained 10 μl of Easy Fast PCR Mix Buffer (TransGen, Beijing, China), 0.5 μM each primer, 10 ng of DNA template, and Milli-Q water to the final volume. Thermal cycling was conducted as follows: initial denaturation at 95°C for 5 min, followed by 35 cycles of denaturation at 95°C for 1 min, annealing at 55°C for 30 s, and elongation at 72°C for 30 s, with a final step of 72°C for 5 min. After purification with a GeneJET Gel Extraction kit (Thermo Scientific), all three types of PCR products were sequenced on HiSeq 2500 sequencer (Illumina) (Magigen, Guangzhou, China) using a paired-end approach. The sequences acquired were processed according to the protocols described in previous works [33–35]. Briefly, we used DADA2 (version 1.12.1) to obtain denoised [36], chimera-free, nonsingleton microbial ASVs based on the default parameters. Taxonomic annotation of ASVs was performed using the SILVA SSU 138.1 database for bacteria and the PR2 SSU 4.12.0 database for protists [37, 38]. To focus on protists, we removed the sequencing reads assigned to *Rhodophyta*, *Streptophyta*, *Fungi*, *Metazoa*, unclassified *Opisthokonta*, and ambiguous taxa in eukaryotic community data. For T4-like virus, representative nucleotide sequences of the *g23* gene fragments were translated using MEGA-X [39]. The closest relative of the representative sequences was determined using BLAST analysis [40]. The ASV tables for bacteria, protists, and T4-like viruses were rarefied to the minimum number of sequences per sample. A total of 3 915 528 sequences were produced across three targeting sections of the standard DNA barcoding regions. After quality filtering, 8361, 2564, and 545 ASVs were retained for the bacterial, protistan, and T4-like virus datasets, respectively. On average, bacterial communities were dominated by *Proteobacteria* (36.1%), *Actinobacteria* (24.0%), and *Acidobacteria* (10.8%); protistan communities were dominated by *Rhizaria* (43.1%), *Amoebozoa* (28.1%), *Stramenopiles* (12.4%), and *Alveolata* (9.3%); and T4-like virus communities were dominated by the *Paddy group* (63.9%), *Paddy clones* (17.6%), and *Lake groups* (12.8%) (Fig. S1). Not all of the samples passed our rarefaction cutoff, and we obtained information for 73 out of 76 study sites. The rarefaction curves of passed samples are provided in Fig. S2. This information was used for downstream analysis.

### Geographic distribution patterns of microbial communities

We selected species richness (that is, the number of observed ASVs) as a commonly used biodiversity metric to evaluate the diversity of microbes. Richness is the most frequently used and simplest metric of biodiversity [41]. We tested the relationships (linear or quadratic regressions) between microbial richness and latitude by the “lm” function in R. We identified the best model for the regression by the lowest Akaike information criterion (AIC). Nonmetric multidimensional scaling (NMDS) analysis based on Bray–Curtis distance metrics was performed to explore differences in microbial composition at the ASV level (Hellinger transformed) across latitudes. The dissimilarity of microbial composition in different latitudinal groups was tested by analysis of similarity (ANOSIM) using the “*vegan*” package. Distance-decay relationships (DDRs) were evaluated with ordinary least-squares (OLS) regression between the Bray–Curtis similarity and geographic distance matrices. We tested the slopes of the DDRs in the two distinct latitudinal groups (low- and high-latitudinal groups) to compare the differences between them.

### Quantification of assembly processes of bacterial community

We quantified the importance of deterministic processes of the bacterial community using the “1—normalized stochasticity ratio (*NST*)”. *NST* is an index developed to estimate the ecological stochasticity in the community assembly process [with 50% as the boundary between more deterministic (<50%) or more stochastic (>50%) assembly] [42]. By considering the overall performance of similarity metrics, the *NST* based on Jaccard distance is recommended for estimating the contribution of stochasticity in community assembly. This analysis was conducted in the “*NST*” package. We used a moving window analysis to quantify the variation in the bacterial assemblage across latitudinal groups. Moving window analysis is a prominent method of analyzing the spatial variability of landscape patterns at multiple scales [43, 44]. For each focal unit in the landscape, a matrix is used to specify the neighborhood, and the metric value of this local neighborhood is assigned to each focal unit [45]. Therefore, the windows are allowed to overlap and the result of a moving window analysis is a raster with an identical extent as the input [44]. However, each unit describes the neighborhood in regard to the variability of the selected metric [43]. Here, we selected the low-latitude group as the first unit by continuously including sampling points at higher latitudes while removing sampling points at lower latitudes, such that the low-latitude group transitioned to the high-latitude group.

### Bipartite networks

To evaluate the associations of bacterial, protistan, and T4-like virus communities, we constructed a predator–prey bipartite network based on the ASV relative abundance datasets. Bipartite networks are the representation of relationships between two distinct classes of nodes, such as predator–prey, plant–pollinator, and parasite–host interactions [46]. Identifying patterns in bipartite networks is useful in explaining the formation and function of putative trophic interactions [46, 47]. We constructed bipartite qualitative (binary) networks using the R package “*bipartite*” v.2.08 and visualized in Cytoscape v.3.9.0 [48, 49]. In order to retain more information and reduce computational complexity, only ASVs detected over 30% of sampling sites were kept for the meta-network (of 73 samples) construction. The selection of the threshold of 30% is based on previous studies [50, 51], and our analysis confirmed that the choice of threshold did not affect our main findings (Fig. S3). For instance, the latitudinal patterns of network metrics were consistent at the filtering thresholds of 20%, 30%, and 40%. Moreover, the network metrics along latitudinal gradients were significantly correlated with each other in filtering threshold of 20%, 30%, and 40% (Fig. S3). Before constructing the bipartite network, the filtered table was used for pairwise correlation calculation of predators (protist and T4-like virus) and prey (bacteria) using Spearman rank correlations. This was followed by an Random Matrix Theory (RMT) based approach to automatically determine the correlation cutoff threshold, implemented using the “*RMThreshold*” package [52]. This RMT-based approach avoids an arbitrary transition point (St) determination commonly used in association-based network methods, thus minimizing the uncertainty in network construction and comparison [53]. The obtained adjacent matrix associated with the bipartite graph was generated, which filtered the noncorrelated associations and consisted of 1 or 0, showing the presence/absence of the corresponding predator–prey association [46]. To explore the latitudinal patterns of associations of bacteria, protists, and T4-like viruses, a subnetwork of each sampling point was constructed using the “subgraph” function in the “*igraph*” package. The architectures of the observed protist–bacteria and virus–bacteria networks were calculated using the “*bipartite*” package. At the meta-network level, the node number, edge number (total links between nodes in the network), and network connectance (*C* = *E*/*N*^2​^ where *E* is the number of edges, and *N* is the number of nodes) were calculated and summarized (Table S3). At the subnetwork level, edge number and network connectance were selected to evaluate the species associations [54].

### Procrustes analysis

To explore the congruence between bacterial communities and predatory (protists and T4-like viruses) communities across latitudes, we used Procrustes analysis to transform the first two coordinates of the NMDS plot for each microbial community across latitudes with the Bray–Curtis dissimilarity metric [55]. Procrustes analysis is a technique for comparing the relative position of points (i.e. samples or sites) in two multivariate datasets (in an ordination space). It attempts to stretch and rotate the points in one matrix, such as points obtained from an NMDS, to be as close as possible to points in another matrix, thus preserving the relative distance between points within each matrix [55, 56]. This procedure yields a measure of fit, *R*^2^, which is the correlation in a symmetric Procrustes rotation. Analogous to a Mantel test, Procrustes analysis is particularly used to determine how much variance in one matrix (i.e. bacteria) is attributable to the variance in the another (i.e. protists) matrix or to assess the statistical significance in the correlation between the two multivariate datasets. In addition, Procrustes analysis has the advantages of the application of the Procrustean association metric (i.e. residuals). Pointwise residuals indicate the difference between two different community ordinations for each sample and are used to examine predator–prey associations across latitudinal gradients. The statistical significance of the Procrustes analysis (i.e. *R*^2^) can be assessed by randomly permutating the data 1000 times [57]. This analysis was performed using the R package “*vegan*” v.2.4.6.

### Multiple factors impact bacterial community diversity and composition

To explore the impact of environmental effects (climatic and edaphic factors) and biotic effects (P–B and V–B associations) on bacterial α-diversity, we used multiple OLS regression to analyze the relationship between the potential explanatory variables and species richness. The best models were identified based on the lowest Akaike’s information criterion (AIC) [58]. OLS was performed using the function “lm” in the “*car*” package.

Redundancy analysis based on Bray–Curtis distance (dbRDA) was performed to reveal the effects of environmental factors versus biotic associations on the bacterial community [59]. Prior to dbRDA, the attributes were manually selected according to variation inflation factors (VIFs), resulting in VIFs < 10. The statistical significance of each explanatory variable was examined with a permutation test (999 random permutations), and only significant variables were retained (Table S4). To quantify the relative contribution of the environmental effects (climatic and edaphic impacts) and the biotic effects (P–B and V–B associations) on bacterial β-diversity across the latitudinal groups, we adopted a moving window analysis combined with hierarchical partitioning method using the “rdacca.hp” function in the “*rdaaaca.hp*” package [60]. The independent effects correspond to their relative contribution to the total variation. Similarly, we used Mantel tests to determine the correlation between community Bray–Curtis distances (protist–bacteria and virus–bacteria) from low to high latitudinal groups.

To disentangle the direct and indirect relationships of environmental and biotic effects on bacterial richness and community structure at three spatial scales (total sites, low latitudinal group, and high latitudinal group), random forest analysis [61] and partial least squares path modeling (PLS-PM) [62] were constructed using “*randomForest*” and “*plspm*” packages, respectively. The bacterial community structure was represented by NMDS1 of NDMS analysis based on Bray–Curtis distance. We first considered a full model that included all reasonable pathways, and then, we eliminated nonsignificant pathways until we obtained the final model whose pathways were all significant. To reduce the model complexity, we constructed composite variables for climatic factors (MAT and MAP), edaphic factors (pH, SWC, and C/N), and biotic associations (edges and connectance between protists/virus and bacteria) (Tables S5–S7). Goodness of fit (GOF) statistics were used to measure the model’s predictive power. We also performed VPA to verify the results of PLS-PM [63], and the individual *R*^2^ represents the total effects of climatic factors, edaphic factors, and biotic effects on bacterial communities.

### Microcosm experiment

The microcosm experiment was conducted in soils independent of the large-scale survey presented above to assess the relationships between microbial diversity and climatic factors and enable us to explore the latitudinal patterns of biotic associations between bacterial, protistan, and T4-like virus communities independently of the data used. In May 2021, paddy soil for microcosm construction was collected from Guangdong (22.45°N, 112.41°E) in southeastern China. Soil samples were collected from the top 20 cm layer. The local temperature at the time of sampling was 20.7°C. The percentages of soil water content and soil organic matter were 13.7% and 1.4%. The value of pH, NH_4_^+^-N (mg kg^−1^), NO_3_^-^-N (mg kg^−1^), and AP (mg kg^−1^) (measured as described above) were 6.5, 4.1, 82.9, and 39.4, respectively.

For microcosm preparation, 20 g of soil was added to a serum bottle. Five temperature gradients (5°C, 10°C, 15°C, 20°C, 25°C) and four soil water content gradients (10%, 15%, 20%, 25%) were set up to prepare soil microcosms. For the temperature gradient microcosms, we uniformly adjusted the soil water content to 13.7%. For the soil water content gradient microcosms, the incubation temperature was uniformly set at 20°C. We set eight replicates for the five temperature gradients and four soil water content gradients experiments, with a total of 72 microcosms incubated in a sterilized incubator for 4 weeks. The soil water content was maintained by adjusting the weight of serum bottle with addition of sterilized water every 3 days during the incubation period. After incubation, we extracted soil DNA and performed high-throughput sequencing to evaluate the variation in diversity and biotic association of microorganisms along the temperature gradients and soil water content gradients. To guarantee methodological consistency, the sequencing platform and analysis pipeline for the microcosm experiment are same as the field investigation.

## Results and Discussion

### Latitudinal patterns of microbial communities and drivers of bacterial assemblage

We used amplicon sequencing [16S rRNA genes, 18S rRNA genes, and major capsid protein-encoding gene (*g23*) markers] and assessments of climatic conditions and soil chemistry to explore how putative trophic interactions (P–B and V–B associations) and abiotic parameters affect the bacterial community along a latitudinal gradient (Fig. 1a). Bacterial richness declined gradually toward high latitudes (Fig. 1b), confirming the expected latitudinal diversity gradient (LDG), a decline in species richness from the tropics to the poles [64]. Comparably, the species richness of protists was also lowest at the high latitude, but peaked at the intermediate latitude of approximately 32°N (Fig. 1c). However, the richness of T4-like viruses showed a nonsignificant trend toward high latitudes (Fig. 1d). There was a clear clustering of these three taxonomic groups, showing distinct variations in the microbial community composition at low and high latitudes (ANOSIM statistic: *P* < .001) (Fig. 1e–g). We tested the correlation of microbial diversities with environmental parameters and found different response patterns in these three groups (Table S8). Despite MAT, MAP, pH, and SWC significantly affect the richness of three types of microbes; variations in their correlation with C/N and inorganic nitrogen content underscore the critical role of soil nutrient status in influencing speciation rates and community diversity [65]. The distance–decay relationship (DDR) showed a sharper decrease in the compositional similarity of bacteria and protists at low latitudes than at high latitudes, which is contrary to the patterns of T4-like viruses (Fig. S4). These results indicate that the contrasting latitudinal diversity patterns in bacteria, protists, and T4-like viruses may result from their differential responses to environmental filters and dispersal limitations [65, 66].

**Figure 2:**
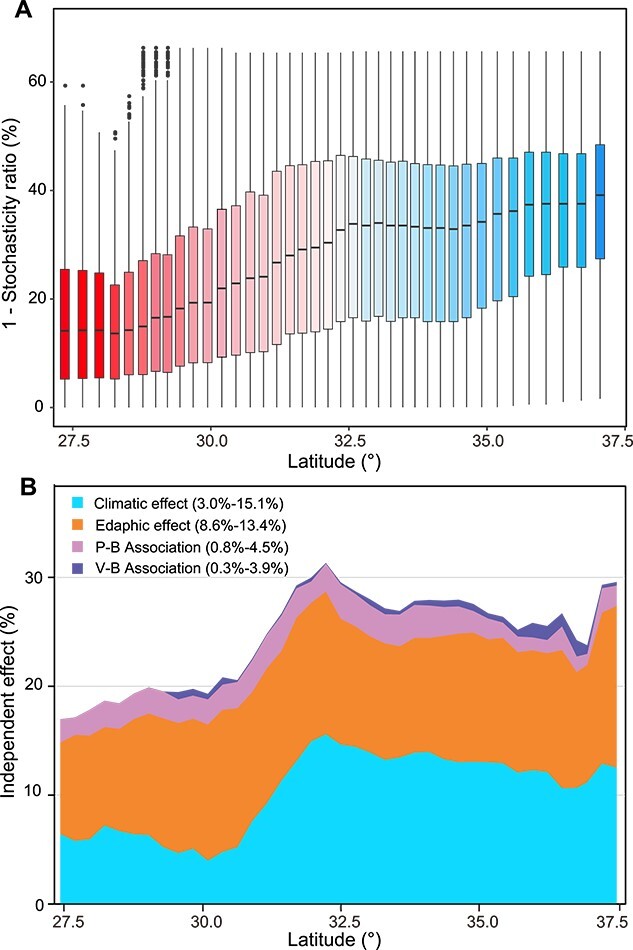
The bacterial community assembly processes and the influence of abiotic and biotic effects on bacterial communities across different categories across absolute latitude. (a) The relative contribution of deterministic processes for bacterial community assembly from low to high latitudes. Deterministic processes of the bacterial community were shown with “1—normalized stochasticity ratio (*NST*).” (b) the relative importance of climatic (mean annual temperature-MAT, mean annual precipitation-MAP, min temperature of coldest month-MTCM, and precipitation seasonality (coefficient of variation)-PSCV), edaphic factors (pH, SWC, and C/N), P–B associations (P–B edges and P–B connectance), and V–B associations (V–B edges and V–B connectance) in predicting the bacterial community structure from the low latitudes group to the high latitudes group.

To illustrate the underlying processes that drive bacterial community assembly from low to high latitudes, we defined the relative contribution of deterministic processes using a moving window analysis (see Methods) [43, 44]. The proportion of deterministic processes gradually increased toward high latitudes until reaching a plateau at 32°N (Fig. 2a). We then quantified the extent to which each independent deterministic effect (including climatic, edaphic, and biotic effects) explained the distribution of the bacterial community (Fig. 2b) [42, 43, 60]. The most important abiotic factors determining bacterial diversity were selected to represent the climatic (mean annual temperature: MAT, and mean annual precipitation: MAP) and edaphic (pH, soil water content: SWC, and C:N ratio: C/N) effects. For the biotic effect, we constructed binary bipartite networks to profile putative top–down controls by protists and T4-like viruses on bacteria and extracted the edge number and network connectance of subnetworks to represent the biotic association of each site (Figs S5 and S6). The consistent latitudinal variation in the proportion of the deterministic processes with the overall size of deterministic factors indicates that both abiotic and biotic relationships influenced deterministic processes (Fig. 2). In most cases, climatic and edaphic effects played dominant roles in structuring the bacterial assemblage. The influence of edaphic factors showed minimal variation across the latitudinal gradient, whereas the impact of climatic factors significantly increased from 3.0% to 15.1% along the latitude of 30°N to 32°N (Fig. 2b). Furthermore, the putative trophic interactions (P–B and V–B associations) were also essential in shaping the bacterial assemblage, although fewer proportions explained the variation in the bacterial assemblage (0.8%–4.5% and 0.3%–3.9% for P–B and V–B associations, respectively) compared to environmental effects (3.0%–15.1% and 8.6%–13.4% for climatic and edaphic effect, respectively) (Fig. 2b). The impact of protists on bacterial communities peaked at mid-latitudes (approximately 32°N), whereas the contribution of T4-like viruses to the bacterial assemblage increased toward high latitudes (Fig. 2b; Fig. S7). We found that the correlation between protists and bacterial communities was greater than that between T4-like virus and bacterial communities (Fig. S7). This may be caused by the fact that protists graze on a wider range of bacterial species than the selective infections by specific viruses [14]. Additionally, the usage of amplicon sequencing may underestimate the correlation between viral and bacterial communities by focusing on T4-like virus [33, 67]. It is widely acknowledged that deterministic factors are mainly composed of abiotic filtering and biotic interactions [68, 69]. However, the impacts of biotic interactions have been understudied, largely due to the challenges in quantifying these interactions and linking them to community assembly processes. We here incorporated the species associations of protists–bacteria and virus–bacteria into ecological models and revealed the importance of species associations on the bacterial assemblages at continental scale. These findings offer a deeper explanation of the assembly processes of bacterial communities in terrestrial ecosystems.

### Both abiotic and biotic effects modulate bacterial diversity

Multiple OLS and PLS-PM were employed to investigate the abiotic and biotic factors influencing bacterial diversity and community structure, respectively. At the continental scale, the OLS analysis showed that the variation in bacterial richness was best explained by MAT, SWC, protist–bacteria association (P–B edge number), and pH (Table 1). Random forest analysis further proved that MAT and SWC were the top two explanatory factors (*P* < .05) for the variation in bacterial richness in both low- and high-latitude regions, followed by the protist–bacteria associations and pH (Fig. S8). These findings are consistent with previous studies, which showed that climate factors and pH were crucial in explaining the variation of bacterial richness [70, 71]. The effect of V–B association was nonsignificant, but was necessary to improve the final model’s fit in high latitudes rather than in low latitudes (Table 1). This may be because that higher enzymatic activity degrades viral capsids in warmer and humid soils, resulting in a lower impact of viruses on bacterial diversity at low latitudes [72–74]. Given that T4-like virus richness gradually increases with latitude, it is reasonable to assume that the latitudinal diversity gradients are closely related to the significance of species interactions. Indeed, multiple studies have linked the latitudinal diversity gradient to a presumed gradient in the importance of biotic interactions [16]. For example, the rate of predation of ants on wasp larvae increased toward the tropics along a latitudinal gradient due to the higher ant diversity in the tropics [75]. These findings suggest that an increase in predator richness may lead to more predation on prey, which, in turn, triggers changes in the intensity of species interactions and ultimately affects prey’s diversity [76, 77].

**Table 1:** Relationships between the bacterial richness and potential explanatory variables modeled using multiple OLS regression. The best models were identified using AIC. The spatial autocorrelation in the model residuals was taken into account. All of the variables were standardized (mean = 0; SD = 1) and are displayed with increasing *P* values. The bold text indicates statistically significant correlations.

	Model *R*^2^	AIC	Explanatory variables	*β* weight
Total sites	0.58	152.4	**MAT** ^***^	0.48
			**SWC** ^***^	0.44
			**P–B Edges** ^*^	−0.19
			**pH** ^*^	0.18
			P–B Connectance	−0.15
			V–B Connectance	−0.12
Low latitudes	0.55	75	**MAT** ^***^	0.58
			**SWC** ^*^	0.26
			P–B Connectance	−0.19
			C/N	0.16
High latitudes	0.58	79.2	**SWC** ^**^	0.56
			**MAT** ^*^	0.22
			**P–B Edges** ^*^	−0.21
			V–B Connectance	−0.09
			AP	−0.02

**Figure 3:**
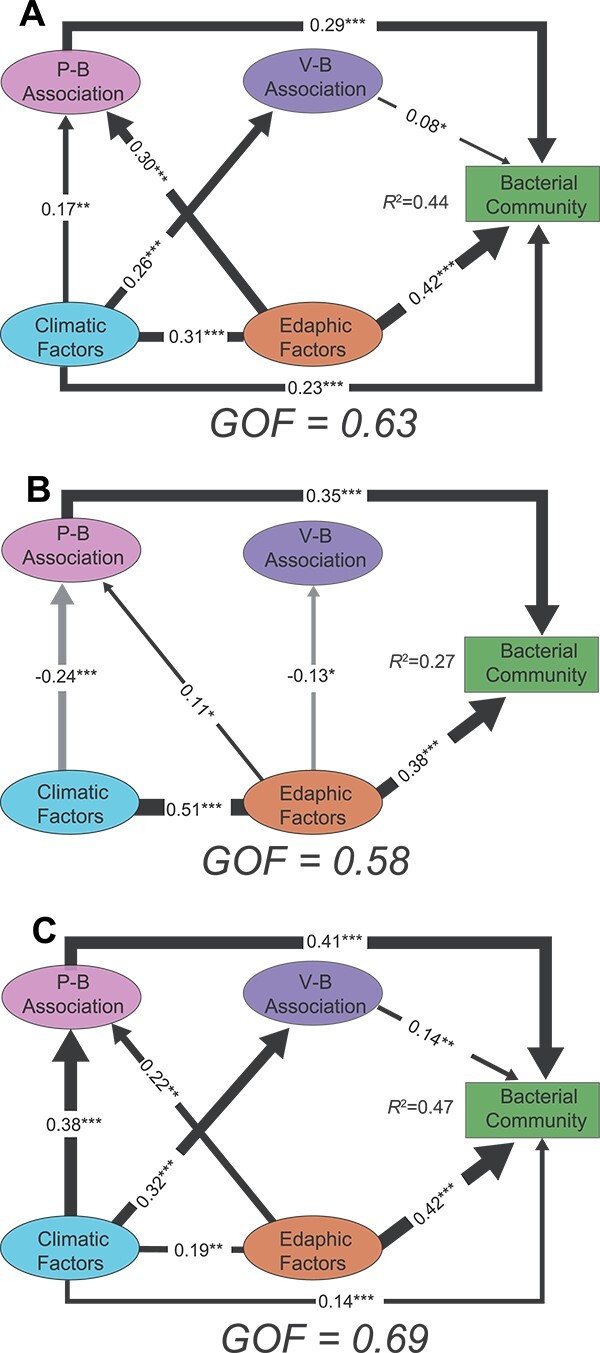
PLS-PMs show the direct and indirect effects of climatic factors, edaphic factors, P–B associations, and V–B associations on the bacterial community structure in all sites (a), the low latitudinal group (b), and the high latitudinal group (c). The composite variables were used to represent climatic factors (MAT and MAP), edaphic factors (pH, SWC, and C/N), P–B associations (P–B edges and P–B connectance), and V–B associations (V–B edges and V–B connectance). Only significant (*P* < .05) relationships are displayed and the width of the arrow is proportional to the strength of the path coefficients. Dark and gray lines represent positive and negative adjusted path coefficients, respectively. *R*^2^ denotes the proportion of the variance explained. Using goodness of fit (GOF) statistics to assess the models’ prediction performance.

We further conducted partial least squares path modeling (PLS-PM) analysis to investigate the associations between abiotic and biotic effects and bacterial community structure across various spatial scales (Fig. 3). Generally, climatic and edaphic factors both directly and indirectly affected the bacterial community at the continental scale in this study, which is in agreement with previous global observations [65, 78]. The effect size of the P–B association (*β* = 0.29, *P* < .001) was greater than that of the V–B association (*β* = 0.08, *P* < .05) (Fig. 3; Figs S9 and S10), which may result from protists grazing on a wider range of bacterial species than specialized viruses [14]. Moreover, focusing on sequencing of *g23* gene fragments rather than directly assessing viruses in soils may also result in a low V–B association [33, 67]. Consistent with the results of variation partitioning analysis (VPA) and Mantel tests (Fig. S11; Table S9), climatic factors had stronger, direct effects on the community structure of bacteria at high latitudes (*β* = 0.14, *P* < .001) (Fig. 3c). Edaphic factors and P–B associations significantly affected bacterial communities, both at the continental and regional scales. V–B associations had a slight effect on the bacterial community on a continental scale (*β* = 0.08, *P* < .05), whereas this effect is stronger in high latitudes (*β* = 0.14, *P* < .01) (Fig. 3). Together, these results indicate that the putative trophic interactions play important roles in modulating the structure of bacterial communities in paddy soils along latitudinal gradients.

### Temperature drives latitudinal patterns of putative trophic interactions

**Figure 4:**
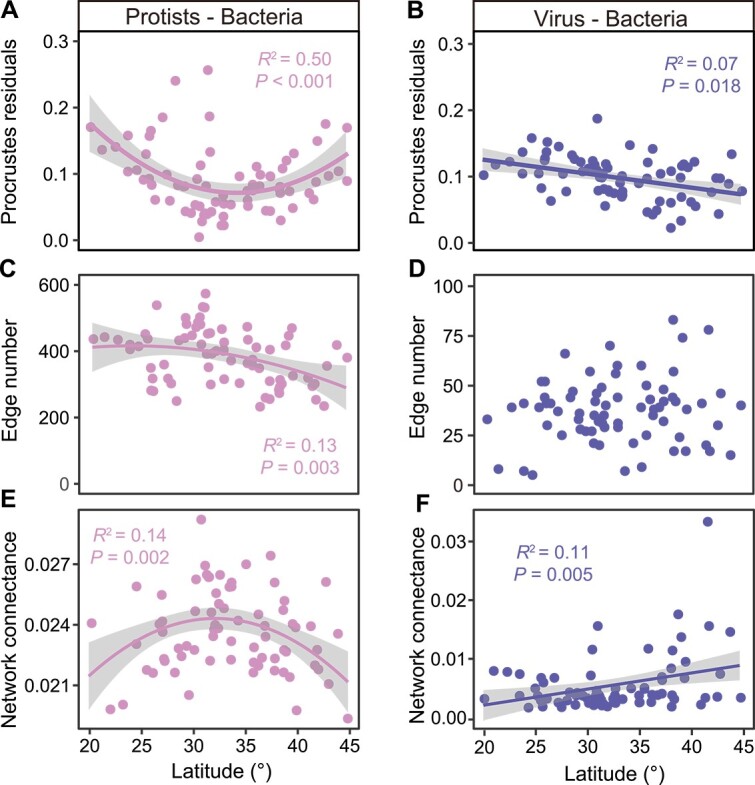
Dynamics of protist–bacteria and virus–bacteria associations across the latitudinal gradient. (a, b) Correlation between the Procrustes residuals and the latitude, the lower values of Procrustes residuals indicate a more congruent composition between bacteria and their predators. (c, d) Correlation between the number of bipartite network edges and the latitude, edge number was estimated using Spearman rank correlation (random matrix theory-based). (e, f) Correlation between the bipartite network connectance and the latitude, the higher values of network connectance indicate stronger interaction. Statistical analysis was performed using OLS linear regressions, and the best-fit model was displayed based on the maximum regression coefficient (the sections with gray shading as 95% prediction interval). The dotted line represents the nonsignificant relationship.

We explored the latitudinal pattern of trophic associations between protists, T4-like viruses, and bacteria at the community and species levels based on Procrustes and bipartite network analysis, respectively. Procrustes is a technique to determine how much variance in one matrix (i.e. bacteria) is attributable to the variance in the other (i.e. protists). Both protistan and T4-like virus communities were strongly associated with bacterial communities at the continental scale, and the associations were clearly separated at 32°N (Fig. S12). The congruence between protistan and bacterial communities (*R*^2^ = 0.75, *P* < .001) was higher than that between T4-like virus and bacterial communities (*R*^2^ = 0.36, *P* < .001), which implies that the impact of protistan predation on the bacterial community is stronger than the impact of T4-like virus infection. Consistent with the latitudinal patterns of microbial diversity and biotic effects described above, the Procrustes residuals followed a quadratic distribution pattern for the P–B association and a monotonically decreasing pattern for the V–B association (Fig. 4a and b). This finding suggests that there are greater putative trophic interactions in the mid-latitudinal zone (~32°N) between protists and bacteria, whereas dominant V–B associations occur in the high latitudinal zone. The edge number and network connectance of the binary networks further support these microbial association patterns, showing the association between protists and bacteria loosens from mid-latitudes to the equator and the North Pole, whereas that between T4-like viruses and bacteria gradually tightens with increasing latitudes (Fig. 4c–f). Numerous studies have shown that the links of network can change with species richness due to the inherent correlation in computational logic [79, 80]. However, the scaling of link numbers is also influenced by evolutionary constraints, phenological matching, and competition [81]. Herein, we found that the latitudinal patterns of P–B and V–B associations are analogous to that of predators’ (protist and virus) diversity but not to that of bacterial diversity (Fig. 1b–d; Fig. 4). A possible reason is that flourishing predation leads to an increase in predator richness and a decrease in prey (bacteria) richness [16], whereas the supply of underlying nutrient resources allows the rapid growth of soil bacteria [82, 83]. We speculated that the stimulation of soil bacteria from the nutrient resources would offsetting the negative effects of predation to some extent, which need more empirical evidence in future.

**Figure 5:**
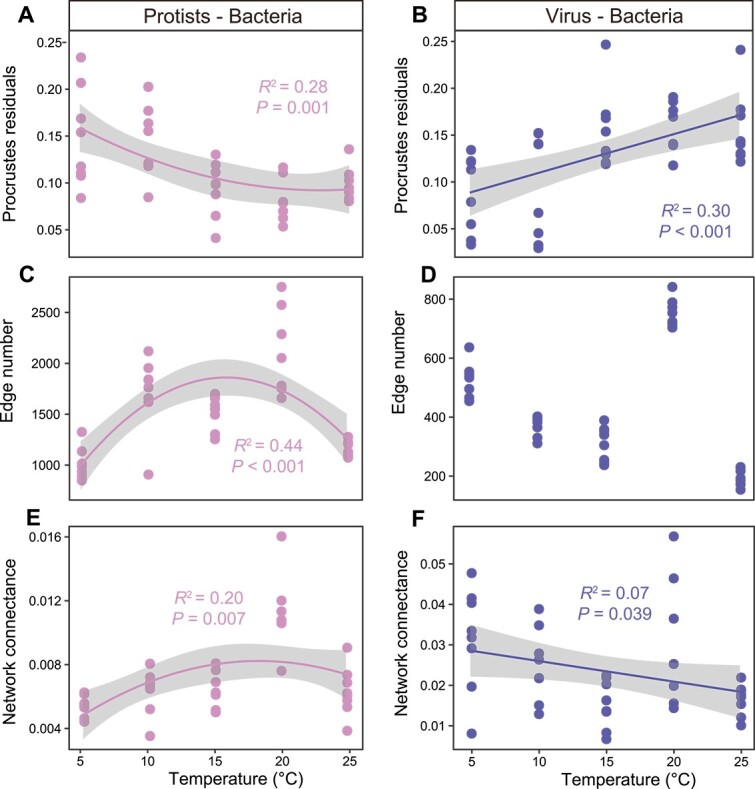
Variation of protist–bacteria and virus–bacteria associations across temperature gradients in the microcosms. (a, b) Correlation between the Procrustes residuals and the temperature. (c, d) Correlation between the number of bipartite network edges and the temperature. (e, f) Correlation between the bipartite network connectance and temperature. Statistical analysis was performed using OLS linear regressions and the best-fit model was displayed based on the maximum regression coefficient (the sections with gray shading as 95% prediction interval). The dotted line represents the nonsignificant relationship.

We found that climatic factors (MAT and MAP) strongly influenced putative trophic correlations between protists, T4-like viruses, and bacteria, as indicated by linear regression analysis (Fig. S13). It is important to note that we examined the community-level relationship dynamics through Procrustes analysis and the species correlations using network analysis. We attributed the correlation patterns to environmental factors only when these patterns consistently align with specific environmental gradients at both community and species levels (Fig. S13). To understand the driving mechanisms of biotic associations along latitudes in depth, we conducted a microcosm experiment to confirm the observed relationships between climatic factors and trophic associations by setting gradients of temperature and soil water content with independent soil samples. A gradient of soil water contents was generated to represent the variation in MAP due to the strong correlation between SWC and MAP at the continent scale (*R* = 0.66, *P* < .001; Table S10). Consistent with the patterns observed in the survey, the microcosm experiment showed that temperature was a crucial factor driving microbial communities (Table S11) and the biotic associations between protists, T4-like viruses, and bacteria (Fig. 5; Figs S14–S17). With rising temperature, the relationship between protists and bacteria initially strengthened and then weakened, whereas the relationship between T4-like viruses and bacteria gradually weakened, as indicated by Procrustes residuals and bipartite network parameters (Fig. 5). The edge number of bipartite networks between T4-like viruses and bacteria exhibited a decreasing trend but was highest at 20°C (Fig. 5d), which is consistent with the observed tendency in network metrics across the latitudinal gradient in the survey. Nevertheless, these results generally showed a consistent pattern of trophic associations with temperatures both in the microcosm experiment and across the latitudinal gradient in the survey.

Compared to temperature, soil water content exclusively affected the association between T4-like viruses and bacteria, and the V–B relationship gradually decreased as the SWC increased (Fig. S18). This trend could be explained by a preference for wetter conditions by dominant bacteria [78]. In turn, these taxa occupied more ecological niches and limited the survival of rare species, thus resulting in lower diversity in microbial communities (Fig. S15). This phenomenon is supported by an analysis of metagenomes in grassland soils with a precipitation gradient, which showed that virus and host diversities are higher in soils with lower precipitation [84]. This higher diversity of viruses and hosts may result in increased predator–prey interactions [77]. Collectively, these results indicate that the latitudinal patterns of putative trophic interactions are predator-dominant and primarily modulated by temperature-related components of climate conditions.

Our data provide empirical information regarding the putative biotic interactions (P–B and V–B associations) underlying the microbial ecological patterns in agroecosystems, these results are novel in revealing the roles of putative trophic interactions in shaping bacterial communities along latitudes. Although our data analyses are rigorous and validated, our sample resources are concentrated in the northern hemisphere at mid-latitudes where human activity is intensive. These findings are complementary to those of previous studies in other ecosystems and together emphasize the specificity and complexity of the mid-latitude region. Recent studies have shown that the distribution pattern of global terrestrial microorganisms is markedly complex in the mid-latitude region of the Northern Hemisphere. For instance, a study found a hump-shaped relationship between the protistan Shannon index and absolute latitude in natural soil ecosystems, with the highest diversity in the 30°N region [85]. Another survey on ocean viral biodiversity identified five distinct ecological zones in the global ocean, with a tendency for viral diversity to decrease first and then increase in the Northern Hemisphere [86]. Furthermore, the temperature changes caused by elevation might complicate our findings on the latitudinal patterns [87]. However, most of our sampling sites (over 80%) are located in low-elevation plains (below 500 m), and there was a weaker correlation between MAT and elevation (*R*^2^ = 0.08, *P* = .017) than that between MAT and latitude (*R*^2^ = 0.83, *P* < .001) across our data. Consequently, elevation variations appear to have a limited impact on our main findings. Future research should consider the combined effects of temperature variations due to both latitude and elevation on species diversity and associations.

We acknowledge that studying viruses using amplicon sequencing only captures parts of the viral community [33, 67], which limits our ability to infer comprehensive virus–bacteria associations based on co-occurrence data. However, the conservation of the *g23*-protein sequenced region generally allows for high-resolution identification of T4-like virus communities [32]. Detection and analysis of virus-bacteria co-occurrence relationships at the high-resolution level offers valuable insights for predicting virus–host dynamics and understanding the evolution of their interactions [88]. Additionally, the direct and indirect top–down controls captured from our observational co-occurrence data may not provide conclusive evidence of biotic interactions [88, 89], and these statistical correlations generate testable hypotheses for future experimental work. Future research could integrate network analysis with experimental approaches in synthetic microbial communities to refine the methods used to evaluate species interactions. Nevertheless, we show the novel influence of biotic associations beyond environmental factors on bacterial communities and discover the drivers of species co-occurrence patterns through microcosm experiments. Our results highlight an important but previously overlooked mechanism of how changing protist–bacteria and virus–bacteria associations that are modulated by climatic conditions could affect bacterial communities along the latitudinal gradients.

## Conclusion

**Figure 6:**
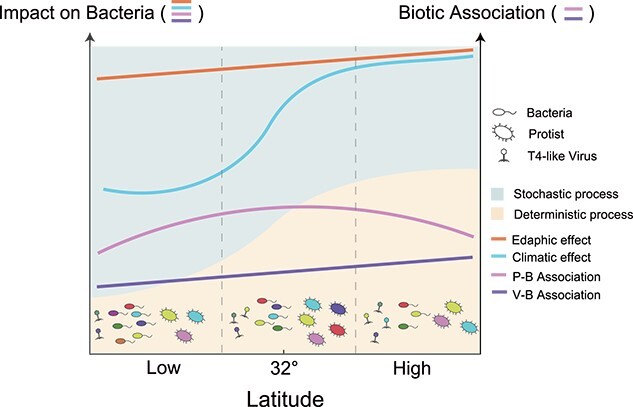
Conceptual paradigm showing the assembly processes for bacterial community changing with latitude in paddy soils. The picture also shows the effects of climatic factors, edaphic factors, and biotic associations (P–B association and V–B association) on bacterial community and the coexistence pattern of protists and bacteria as well as that of virus and bacteria from low to high latitudes. The latitudinal dynamics of the soil microbial diversity is shown at the bottom of the picture.

Combining a large-scale field survey and microcosm experiments, we provided a comprehensive picture of the latitudinal patterns and driving mechanisms of microbial diversity and putative trophic interactions (Fig. 6). We demonstrated that the latitudinal diversity pattern of microbes was kingdom-dependent and depended on the responses of microbes to environmental factors. We found that both environmental factors and biotic associations affected bacterial communities along latitudes, where the intensity of climatic effects sharply increased at intermediate latitude (30°N to 32°N), whereas the intensity of edaphic effects was generally crucial and stable. The putative top–down controls such as protist–bacteria and virus–bacteria associations played a vital role in shaping bacterial structure, although the impacts were less dominant than environmental effects. Furthermore, we provided empirical evidence to prove that latitudinal patterns of putative trophic interactions were primarily modulated by the temperature-related components of climate conditions. These findings enhance our ability to address important ecological theories, as well as to promote soil microbial interconnectedness for performing ecosystem functions and services.

## Supplementary Material

Supplementary_Information_wrae145

Supplementary_Table_wrae145

## Data Availability

The datasets generated during the current study are available in the National Center for Biotechnology Information (NCBI) and National Genomics Data Center (NGDC) repository. Sequences data from the survey experiment have been submitted to the NCBI database with BioProject accession number PRJNA599313. Sequence data from the culture experiment have been submitted to the NGDC under project ID PRJCA022621.
