# Supplementary material for: Temperature-dependent trophic associations modulate soil bacterial communities along latitudinal gradients
Source: ISME J. 2024 Aug 8;18(1):wrae145. doi: 10.1093/ismejo/wrae145 (PMC11334336; doi:10.1093/ismejo/wrae145)
Supplement: Supplementary_Information_wrae145 [file supplementary_information_wrae145.docx]

Supplementary Information for

**Temperature-dependent trophic associations modulate soil bacterial communities along latitudinal gradients**

Xing Huang^1, Σ^, Jianjun Wang^2, Σ^, Kenneth Dumack^3^, Karthik Anantharaman^4^, Bin Ma^1^, Yan He^1^, Weiping Liu^5^, Hongjie Di^1^, Yong Li^1^,*, Jianming Xu^1^

Corresponding author: Yong Li, liyongcn@zju.edu.cn

**This file includes:**

Supplementary results and discussion

Supplementary figures S1-S18

Legends for supplementary tables S1 to S11

Supplementary references

**Other Supplementary Material for this manuscript includes the following:**

Supplementary tables S1-S11

**Supplementary results and discussion**

**Bipartite network characterizations and implications**

In addition to environmental factors, it is well known that biotic interactions play an important role in the origin and maintenance of species diversity. Multiple hypotheses link the latitudinal diversity gradient to a presumed gradient in the importance of biotic interactions [1].

Here, we constructed binary bipartite networks for visualization of putative biotic associations of protists, T4-like viruses, and bacterial ASVs. After significance tests using the Benjamin and Hochberg false discovery rate (FDR) controlling procedure, 552 and 199 possible associations for protist–bacteria and virus–bacteria were statistically significant (*P* < 0.001) (Table S3) and formed one global network (Fig. S5). In the meta-network, 318 bacterial nodes were associated with 92 protistan nodes containing 552 links (467 positive and 85 negative links), and 142 bacterial nodes were associated with 76 viral nodes containing 199 links (154 positive and 45 negative links). These results suggest that protist–bacteria associations are more prevalent than virus–bacteria associations, which may results from protists grazing on a wider range of bacterial species than distinct viral infection [2]. Moreover, 38 bacterial nodes were significantly related to protistan and viral nodes, implying that several bacterial groups were controlled by protozoan grazing and viral infection.

The chord diagram depicts the distribution and the proportion of associations that occurred within networks (Fig. S6). In the protist–bacteria network, there was a great enrichment of connections involving *Proteobacteria* (31%), *Actinobacteria* (22%), and *Acidobacteria* (11%) in bacteria and *Rhizaria* (33%), *Alveolata* (19%) and *Amoebozoa* (18%) in protists, whereas in the virus–bacteria network, *Proteobacteria* and *Actinobacteria* formed a larger proportion (58%) of connections, and the majority of connected T4-like virus belonged to the *Paddy group* (63%) (Fig. S6). It is widely accepted that there is a distinct grazing preference for specific bacterial taxa by protist grazing, while viral infection is distinctly specific [2]. Both *Proteobacteria* and *Actinobacteria* are recognized as the dominant bacterial taxa in soil habitats, and they are also widely preyed upon by protistan grazing and viral infection [3-5]. For example, predation by common soil consumers induces shifts in rhizosphere bacterial community composition, especially several groups in *Betaproteobacteria*, thereby structuring bacteria-plant interactions [6]. In addition, the members of *Firmicutes*, despite having protective cell walls and efficiently resisting viral infection [7], can still be preyed on by protists (Fig. S5) and potentially lead to wilt disease in plants [8]. Although the inference of interacting relationships based on network analysis is somewhat biased, our results provide a glimpse into the study of species coexistence mechanisms.

**Temperature modulates the latitudinal distribution patterns in three microbes**

After four weeks of laboratory incubation, we found that bacterial richness gradually increased with increasing temperature from 5℃ to 20℃, and protistan richness exhibited a hump-shaped pattern along the soil temperature gradient, while T4-like viral richness gradually decreased with increasing temperature (Fig. S14). Moreover, the richness of the three microbes gradually decreased with increasing soil water content (Fig. S15). Nonmetric multidimensional scaling (NMDS) analysis revealed divergent succession trajectories of bacterial, protistan, and T4-like virus communities along with the gradients of soil temperature and SWC (Fig. S14-15; Table S11). As one of the classical theories in the ecological field, the latitudinal diversity gradient (LDG) suggests that organisms have a higher diversity in low-latitude bioregions because they experience greater climate stability than bioregions closer to the poles [9-11]. Organisms inhibiting regions with greater climatic stability are more likely to become specialized in either a particular habitat or a trophic resource because there is sufficient time for divergent selection and speciation to operate [10, 11]. However, it has been found that the taxonomic diversity of bacteria peaked at mid-latitudes and declined toward the poles and the equator, which is contrary to the typical LDG [12]. A possible reason is that environmental heterogeneity drives soil bacteria to exhibit different metabolic demands and in turn affects speciation rates. Additionally, observations have shown rapid growth rates of protists as temperature increased from -1.5℃ to 15℃, while growth rates at the highest temperature (20℃) were less than the maximal rates [13], although the difference was not significant, reflecting a physiological compromise, and a reasonable speculation is that protists are less temperature sensitive at high temperature. Although large-scale studies of soil viral communities are lacking, there is a general downward trend with increasing temperature in warm-temperate marine systems [14]. This relationship, in contrast to the classical LDG, is likely resulted from that soil enzyme activity being higher in warmer and humid environments [15], and in turn degrading viral capsids more strongly [16, 17]. These results suggest that the species’ adaptation to environmental temperature drives their latitudinal diversity gradients.

**Supplementary figure S1-S18**


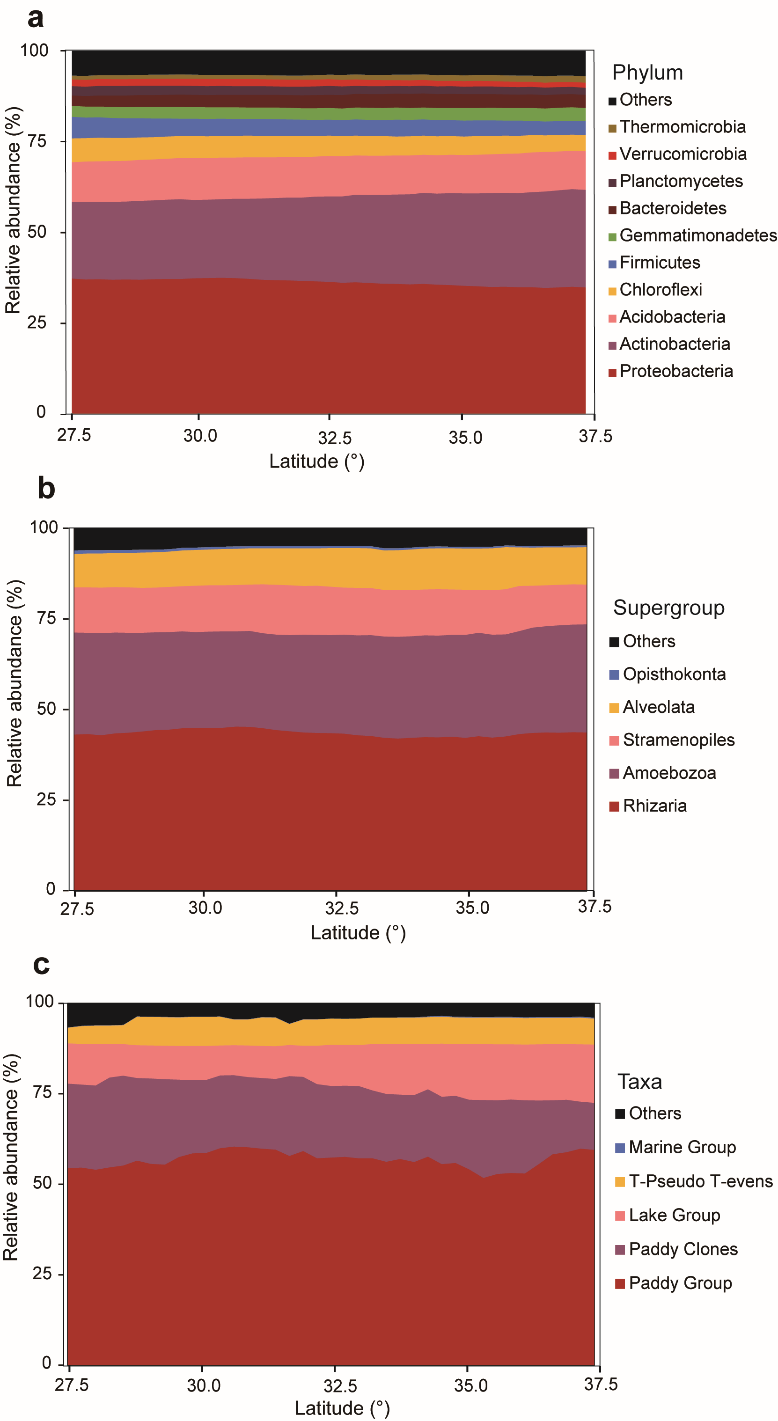


**Figure S1** Stacked barplots showing the relative abundance of taxa for (**a**) bacterial, (**b**) protistan, and (**c**) T4-like virus communities at phylum or supergroup level from the low latitudinal group to the high latitudinal group.


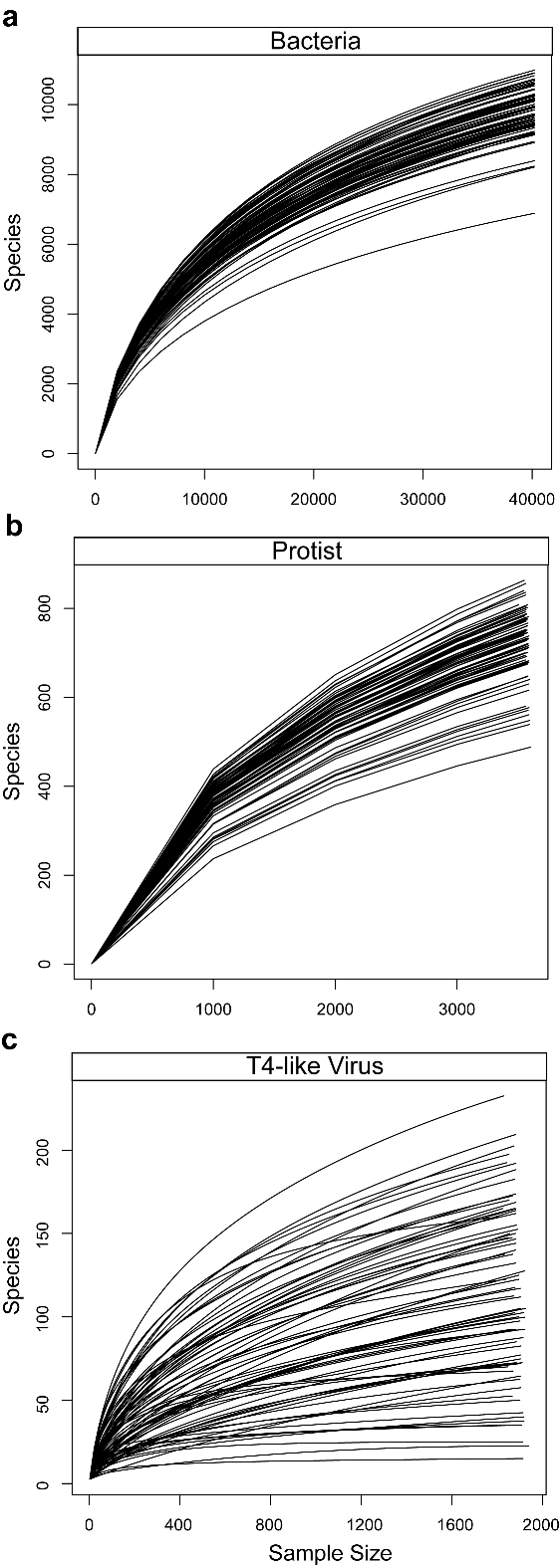


**Figure S2** Rarefaction curves showing observed species richness of bacteria (**a**), protists (**b**), and T4-like viruses (**c**) in samples taken from the 73 sites.

**
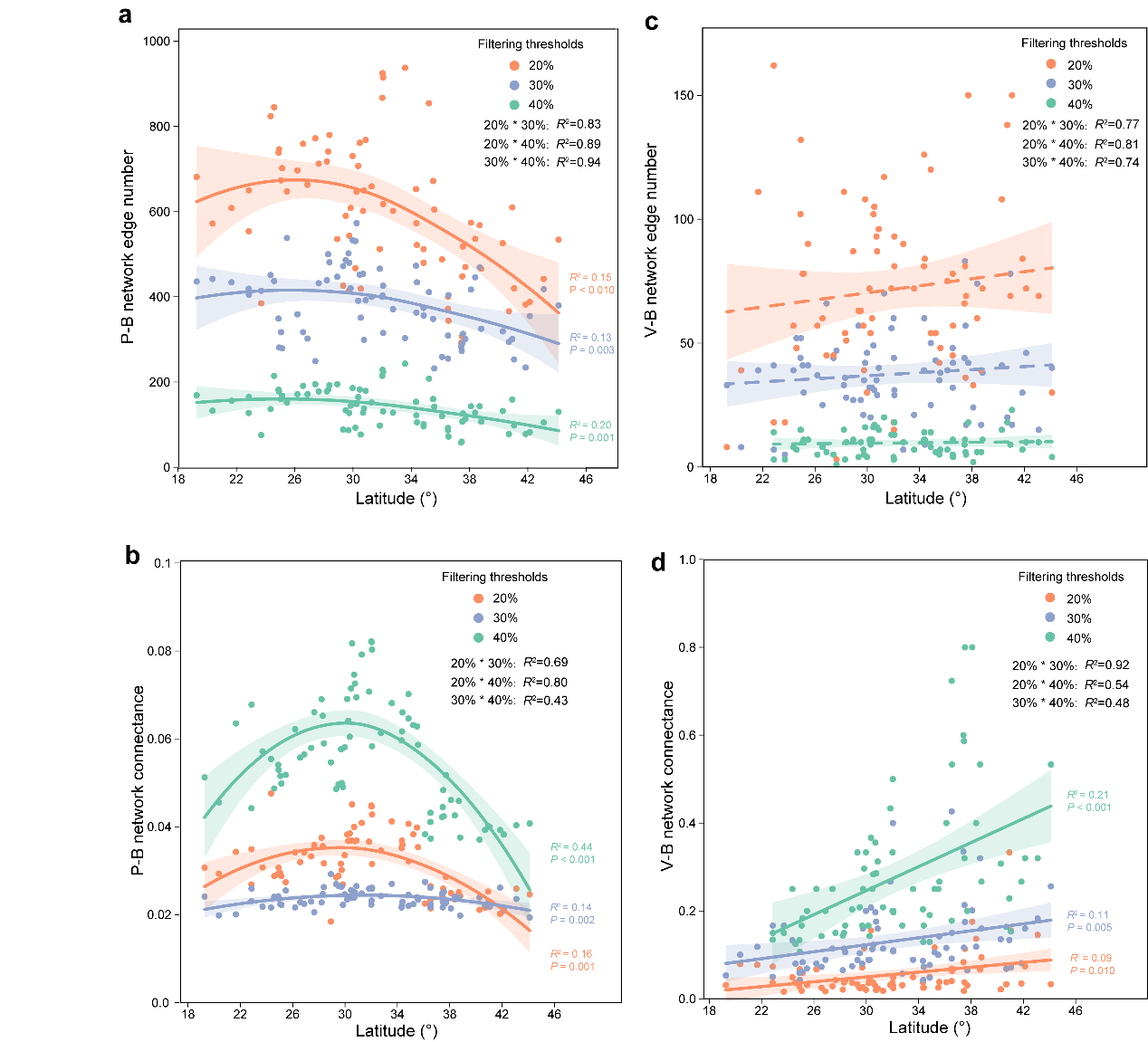
**

**Figure S3** Relationship between network metrics (**a-b,** P-B network; **c-d**, V-B network) and latitude at 20%, 30%, and 40% thresholds of species occurrence frequency. *R*^2^ between different filtering thresholds represents the regression coefficients between network parameters, and the *P* values of all regression coefficients are less than 0.001.

**
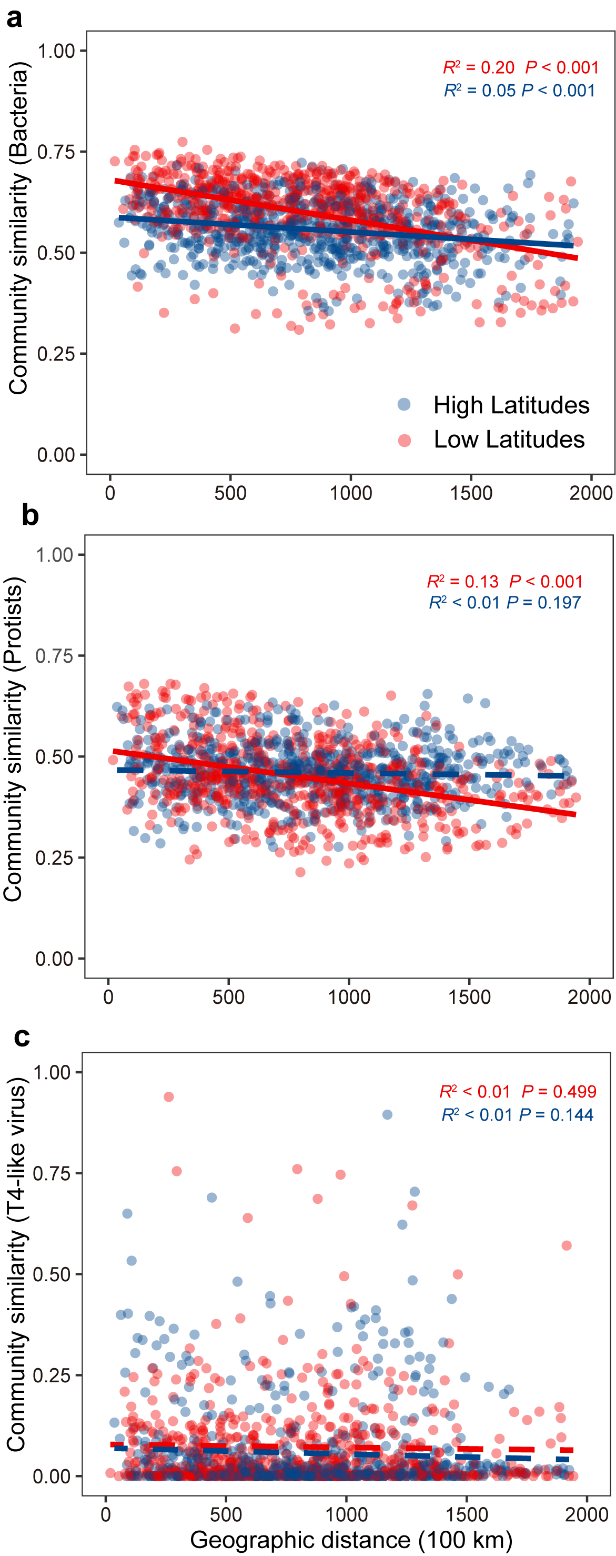
**

**Figure S4** Relationships between the soil microbial (**a**, Bacteria; **b**, Protists; **c**, T4-like virus) community’s similarities (based on the Bray-Curtis distance) and the geographic distance in low and high latitudinal groups.


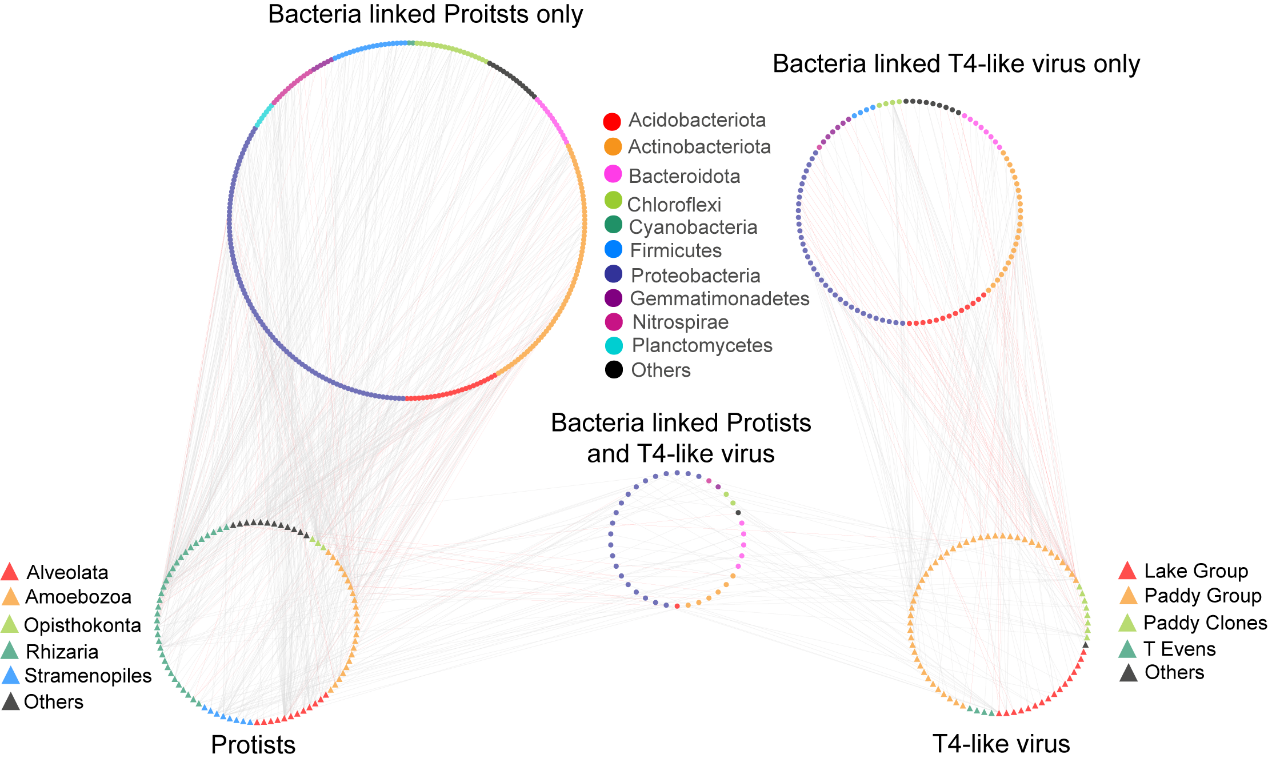
**Figure S5** Overview of the associations include bacteria and their potential predators (protists and T4-like virus). In the network graph, colors of nodes represent different phyla or supergroups. Red links represent negative correlations and gray links represent positive correlations.

**
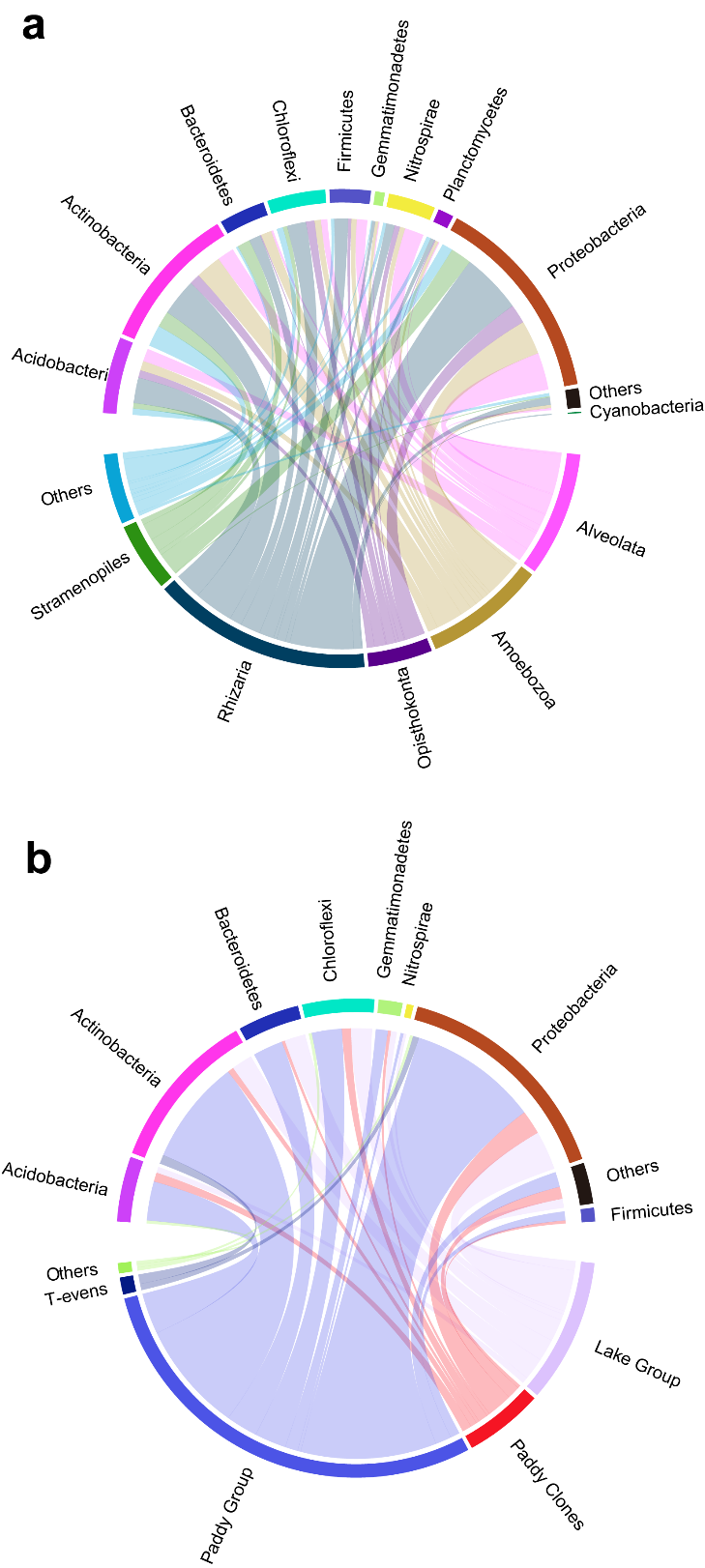
**

**Figure S6** The profiles of bipartite network links among protists-bacteria (**a**) and virus-bacteria (**b**) networks.


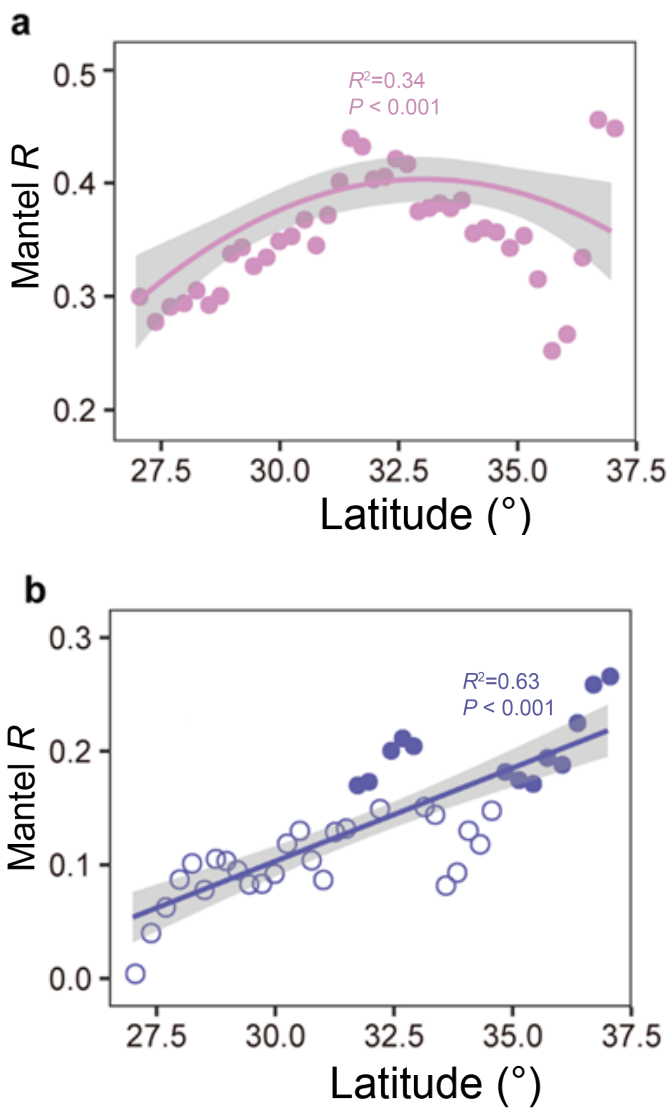


**Figure S7** Dynamics of the impact of protists **(a)** and T4-like virus **(b)** communities on bacterial communities from the low latitudinal group to the high latitudinal group. Dots represent the results of mantel test between protists and bacterial communities as well as that between virus and bacterial communities. Spearman correlation coefficients (*R*) and associated *P* values were calculated. Solid dots represent significant r values (*P* < 0.05), hollow dots represent non-significant *R* values (*P* > 0.05). The microbial dissimilarities were represented by Bray-Curtis distances at ASVs level. Statistical analysis was performed using ordinary least squares linear regressions and the best fit model was displayed based on the lowest Akaike Information Criteria (The sections with gray shading as 95% prediction interval).


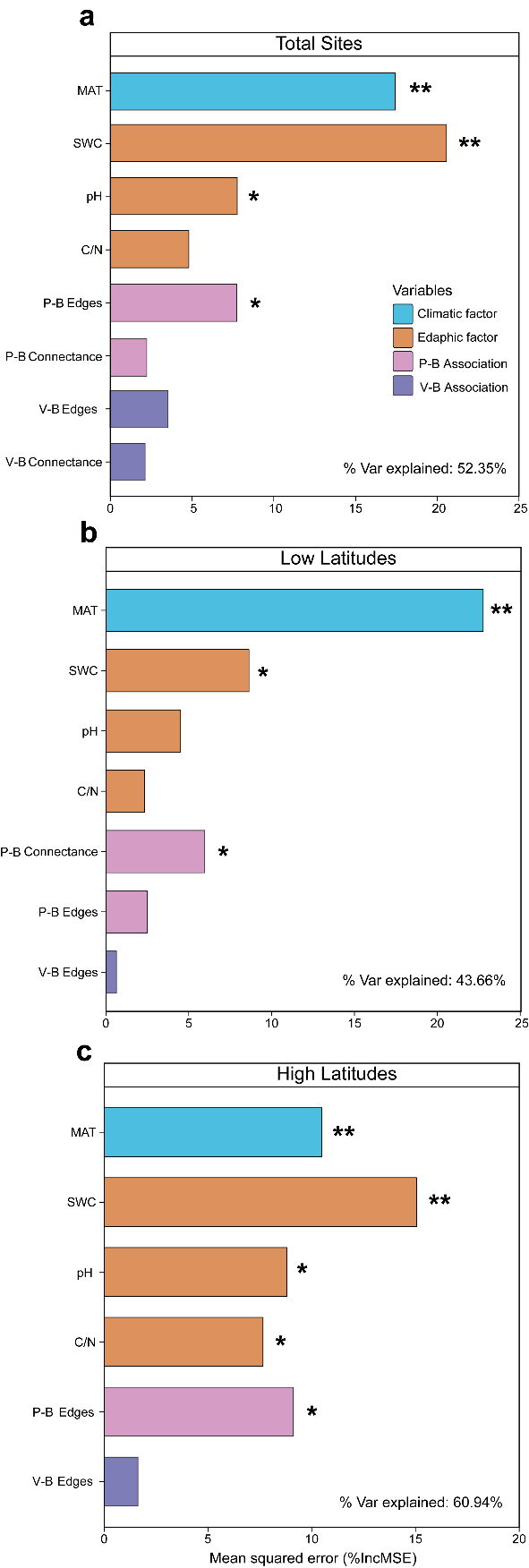


**Figure S8** Potential contributions of abiotic and biotic factors to the bacterial richness in all sites (**a**), the low latitudinal group (**b**), and the high latitudinal group (**c**). The bar plot shows the value of increased in the mean squared error (%IncMSE) of random forest analysis (*, *P* < 0.05; **, *P* < 0.01).


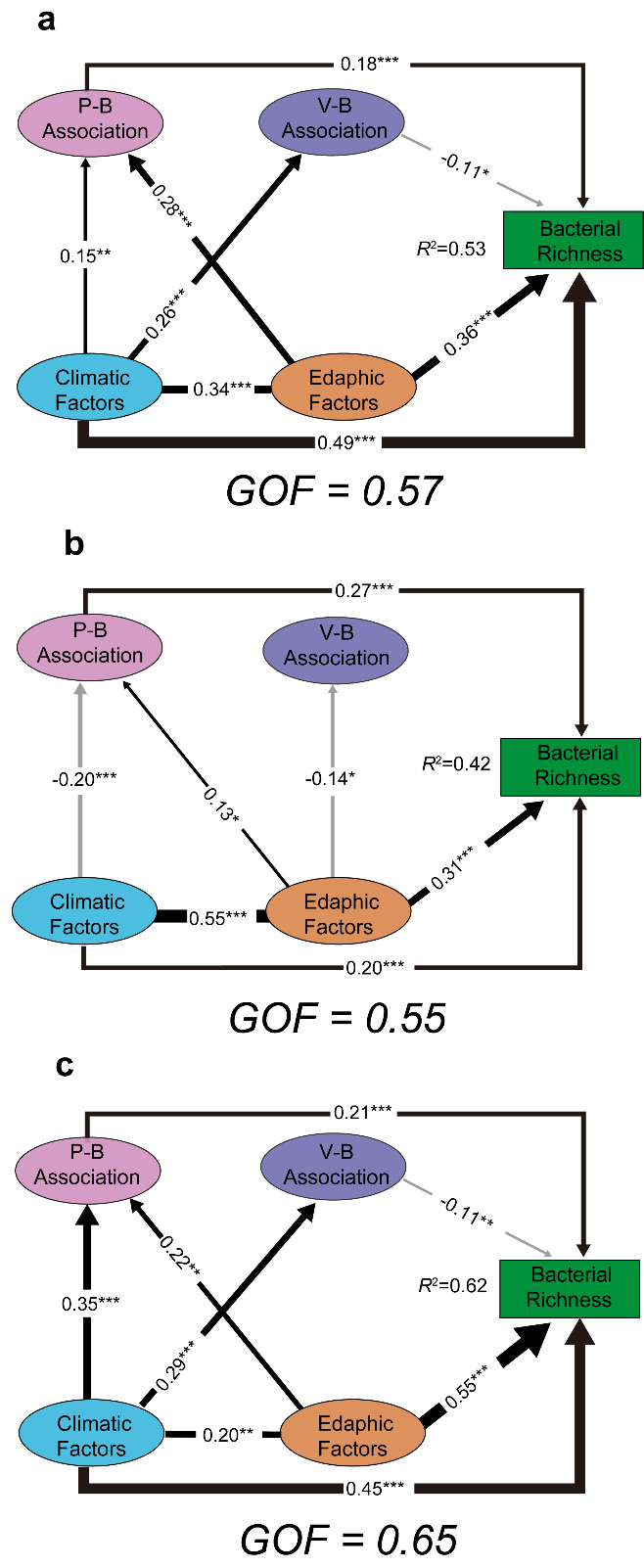


**Figure S9** Partial least squares path models (PLS-PM) show the direct and indirect effects of climatic factors, edaphic factors, P–B associations, and V–B associations on the bacterial diversity in all sites (**a**), the low latitudinal group (**b**), and the high latitudinal group (**c**).**
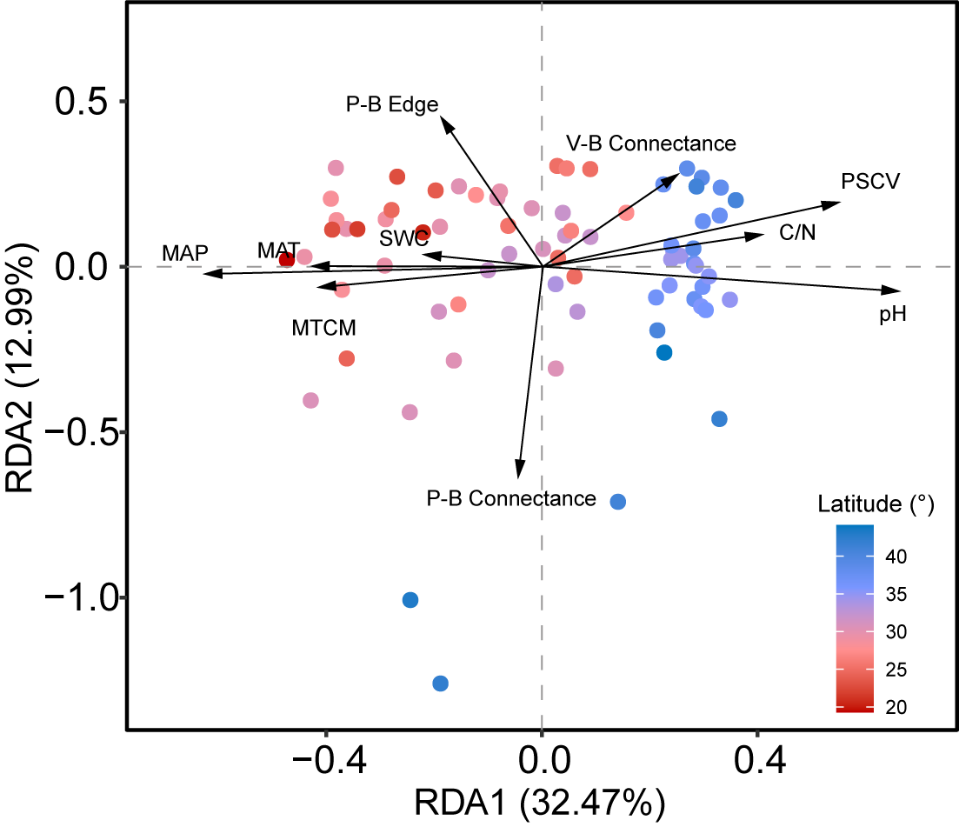
**

**Figure S10** Redundancy analysis (RDA) shows climatic, edaphic factors and biotic effects that influenced bacterial community structure. Sample points are colored according to absolute latitude. The color bar from red to blue represents latitude from low to high. MAT, mean annual temperature; MAP, min temperature of coldest month; MTCM, min temperature of coldest month; PSCV, precipitation seasonality; SWC, soil water content; C/N, C: N ratio; P-B Edge, statistically significant connection between bacteria and protists; P-B Connectance, measures how well-connected all ASVs are to one another in the bacteria-protists network; V-B Connectance, Measures how well-connected all ASVs are to one another in the bacteria-virus network.


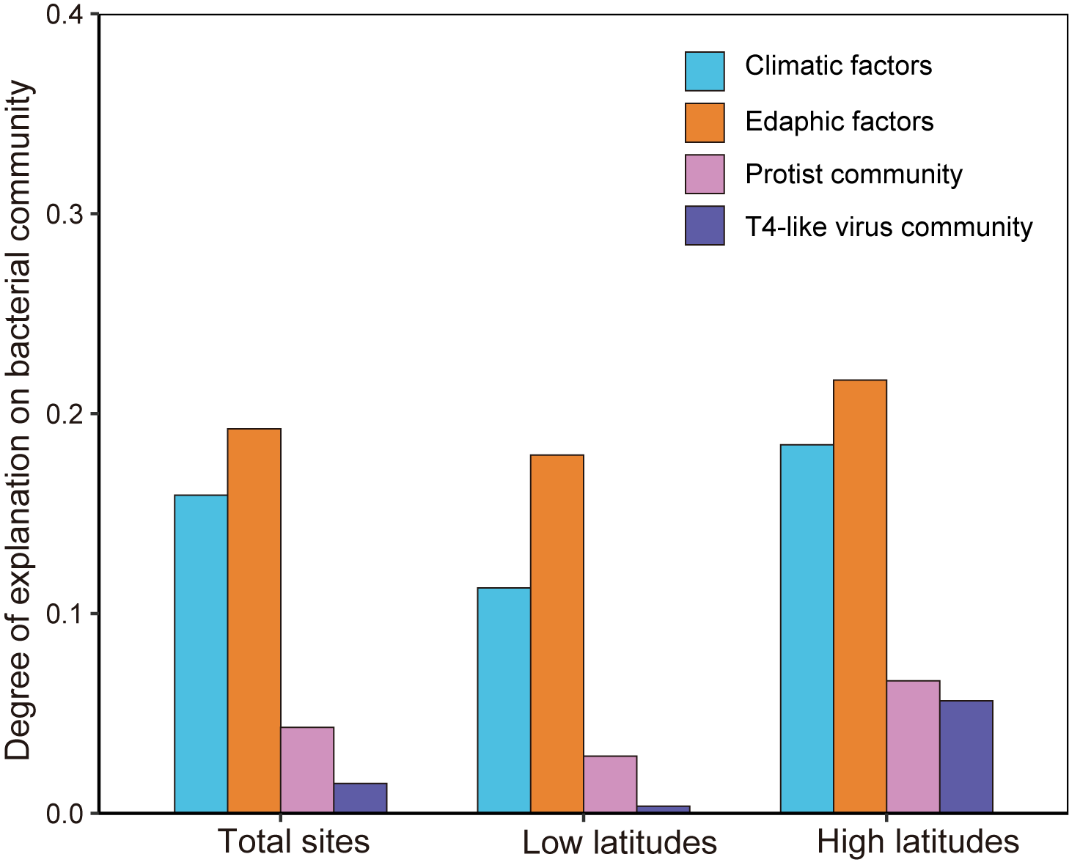


**Figure S11** Variation partitioning analysis of the bacterial community explained by climatic factors (MAT and MAP), edaphic factors (pH, SWC, and C/N ratio), protistan community and T4-like virus community.


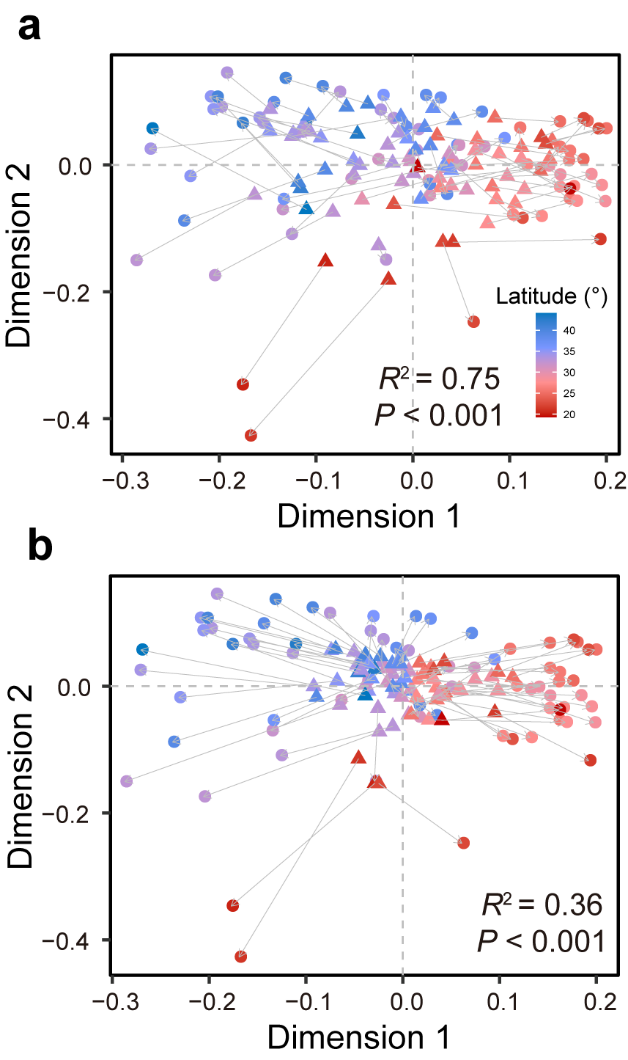


**Figure S12** Procrustes analysis of the correlation between bacteria and protists (**a**) as well as that between bacteria and T4-like virus (**b**) along the absolute latitude based on the NMDS (Bray-Curtis) results. The circles represent bacterial communities and the triangles represent protists or T4-like virus communities. The color bar from red to blue represents latitude from low to high.

**
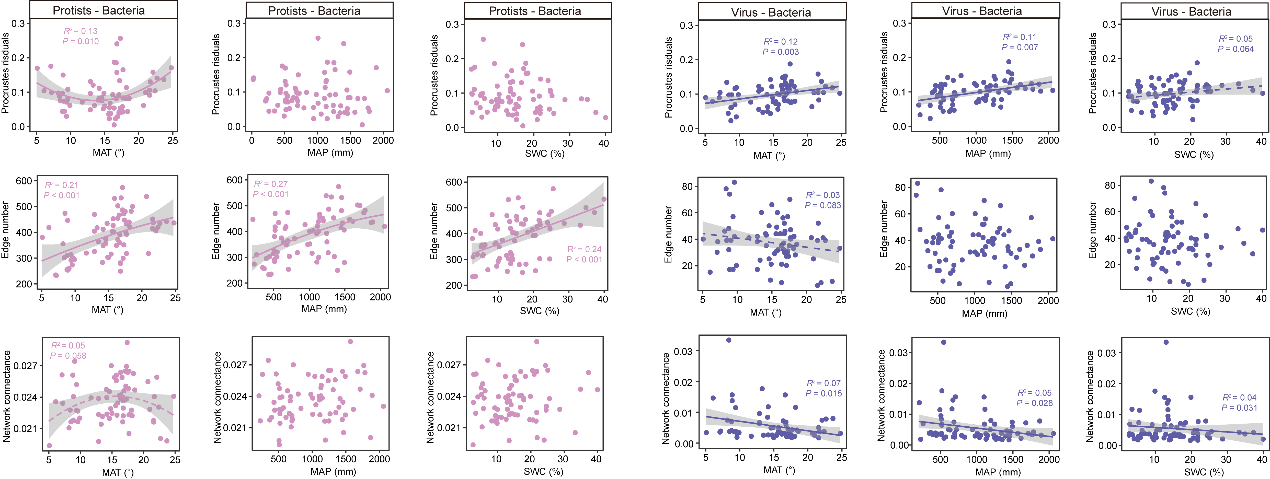
**

**Figure S13** Patterns of the associations between bacteria and predators (protists and T4-like virus) across MAT, MAP, and SWC. The association strength is shown in terms of the number of network edges, the network connectance and the Procrustes residuals. The different colors represent different associations (pink, the links between protists and bacteria; blue, the links between virus and bacteria; *n* = 73). Statistical analysis was performed using ordinary least squares linear regressions and the best fit model was displayed based on the lowest Akaike Information Criteria (The sections with gray shading as 95% prediction interval). The dotted line represents the non-significant relationship.


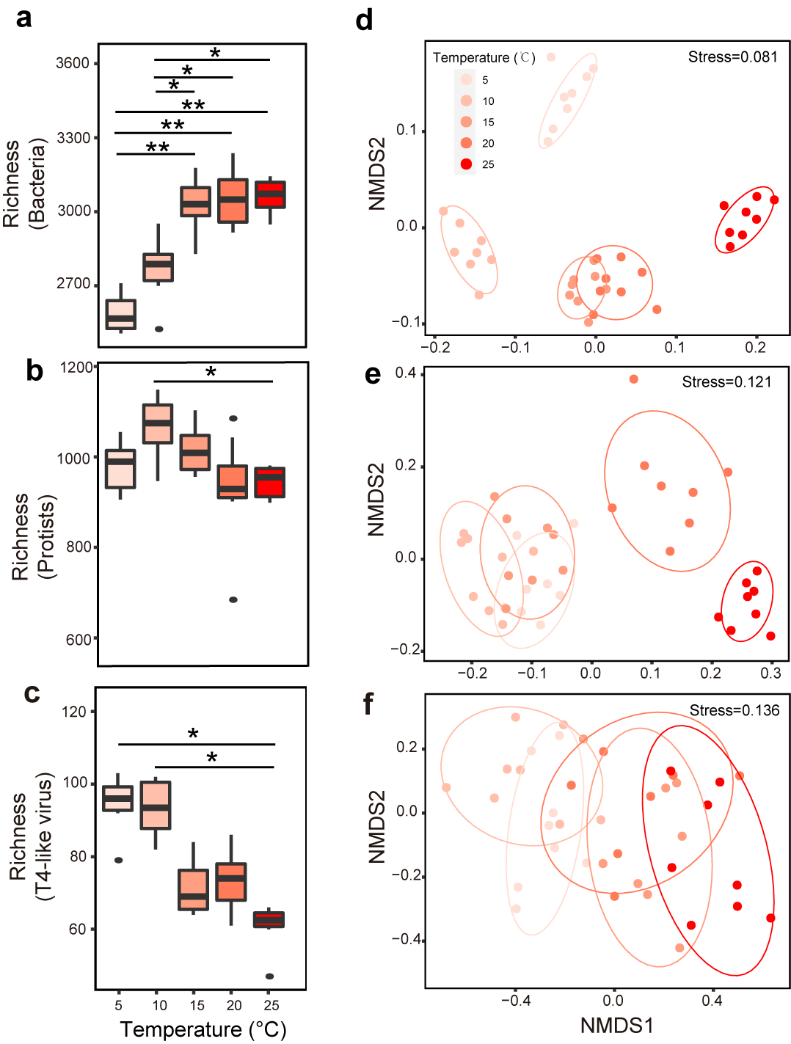


**Figure S14** The diversity and structure of microbiome in microcosm experiments under five temperature gradients. Succession of microbial alpha-diversity **(a-c)** and beta-diversity **(d-f)** under five temperature gradients. The Wilcoxon test was applied, and significant differences observed in a comparison group are labelled with asterisks (**P* < 0.05; ***P* < 0.01; ****P* < 0.001).

**
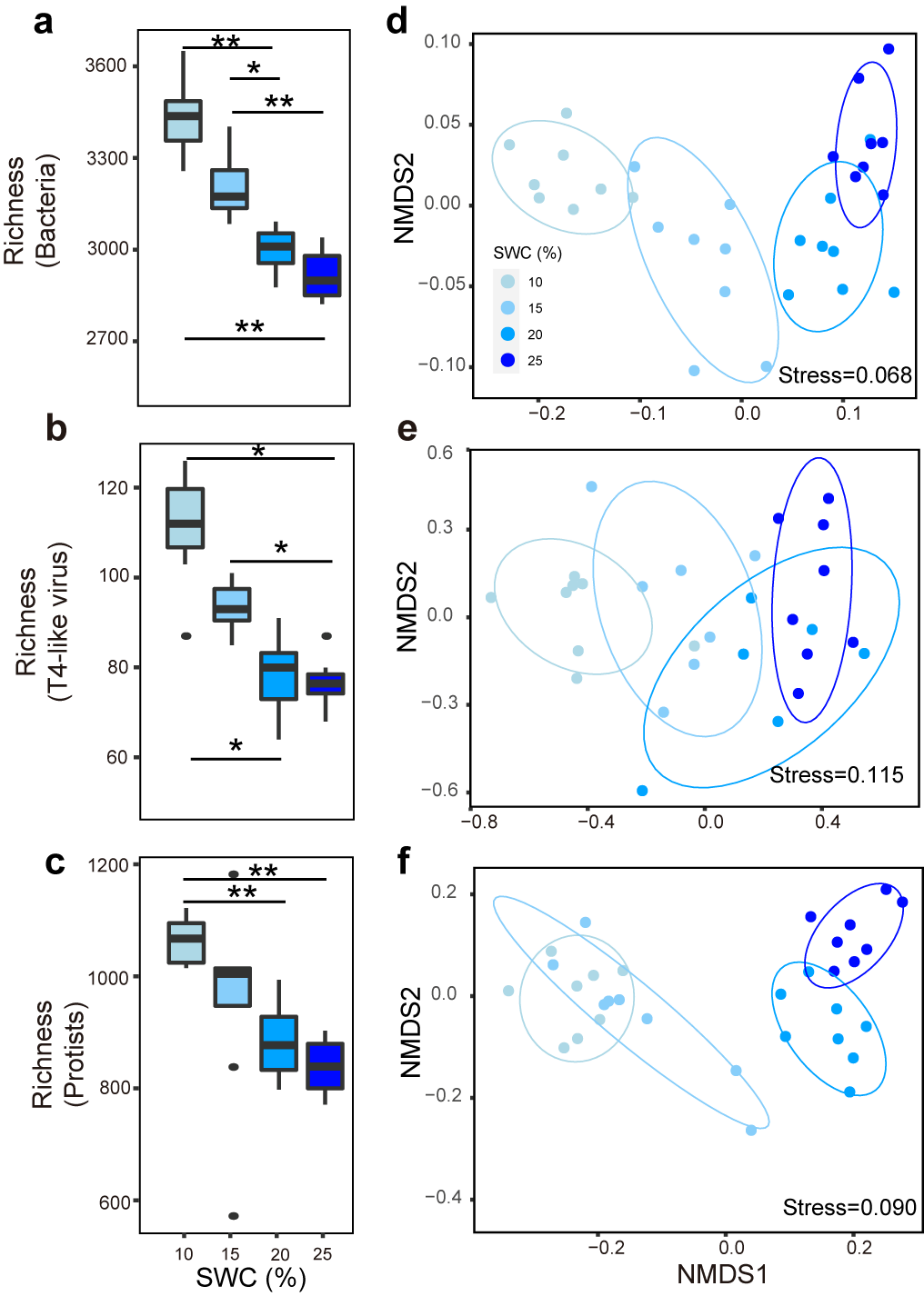
**

**Figure S15** The diversity and structure of microbiome in microcosms under four soil water content (SWC) gradients. Succession of microbial alpha-diversity **(a-c)** and beta-diversity **(d-f)** under four SWC gradients. The Wilcoxon test was applied, and significant differences observer in a comparison group are labelled with asterisks (**P* < 0.05; ***P* < 0.01; ****P* < 0.001).


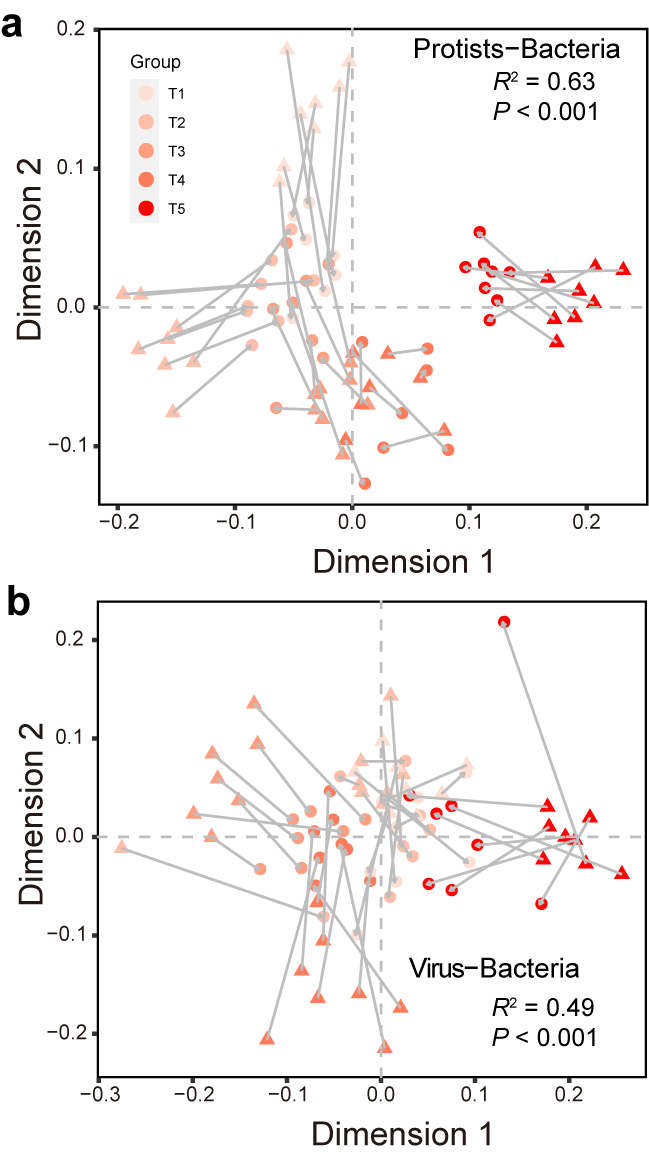


**Figure S16** Procrustes analysis of the correlation between bacteria and protists **(a)** as well as that between bacteria and T4-like virus **(b)** under the five temperature gradients based on the NMDS (Bray-Curtis) results. The circles represent bacterial communities and the triangles represent protists or virus communities.


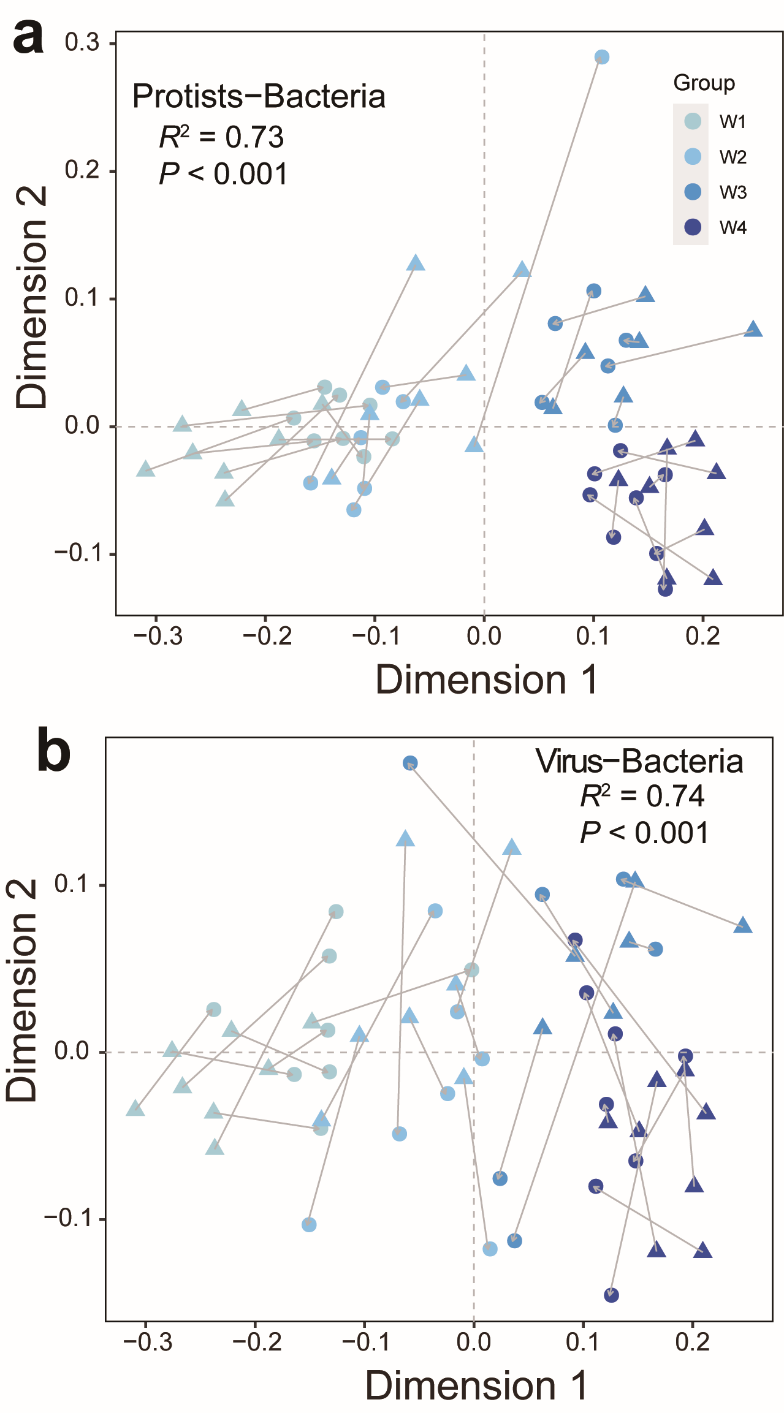


**Figure S17** Procrustes analysis of the correlation between bacteria and protists **(a)** as well as that between bacteria and T4-like virus **(b)** under the four soil water content (SWC) gradients based on the NMDS (Bray-Curtis) results. The circles represent bacterial communities and the triangles represent protists or virus communities.

**
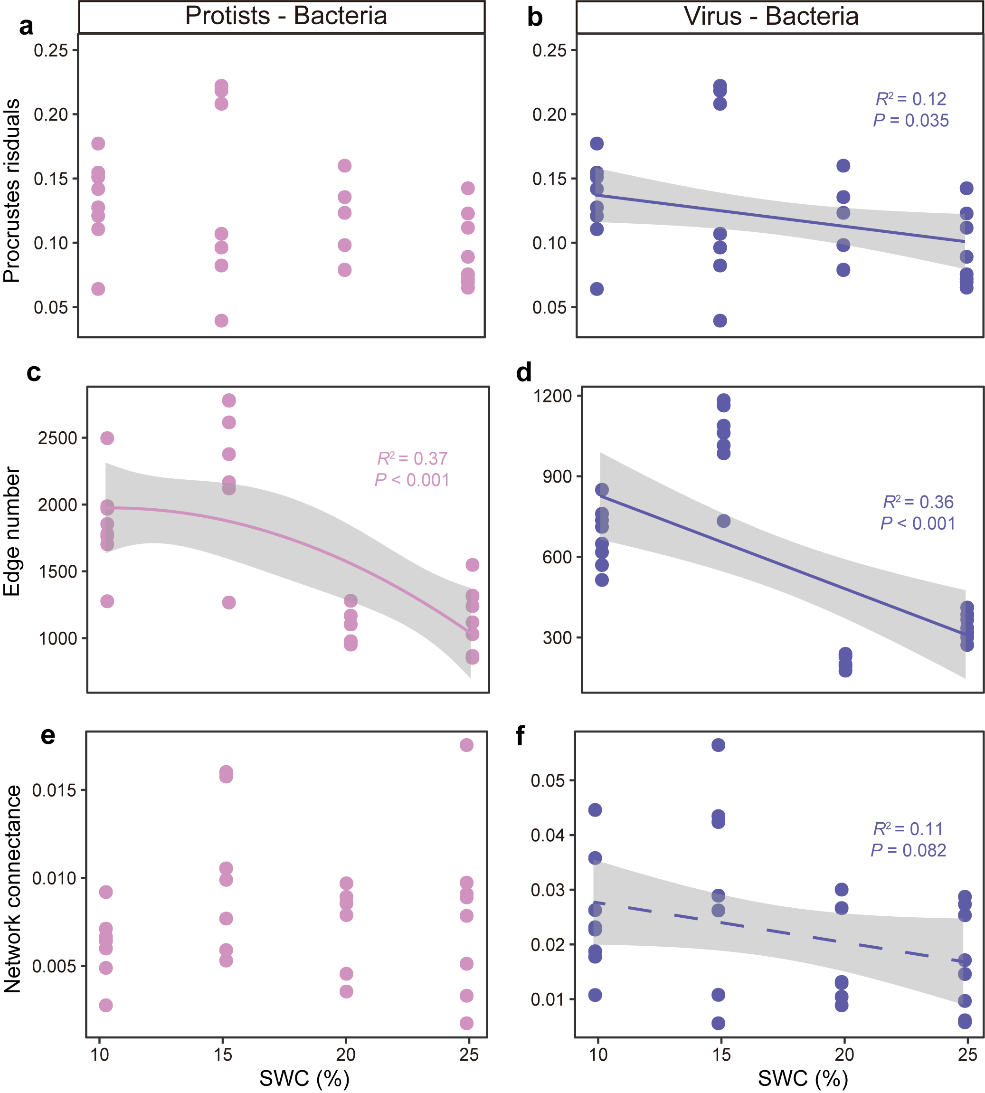
**

**Figure S18** The variation of the associations of protists-bacteria and virus-bacteria under four soil water content (SWC) gradients in microcosmic experiment. **(a-b)** Correlation between the Procrustes residuals and the SWC. **(c-d)** Correlation between the number of bipartite network edges and the SWC. **(e-f)** Correlation between the bipartite network connentance and the SWC. The different colors represent different interactions (pink, the interaction between protists and bacteria; blue, the interaction between virus and bacteria; *n* = 30). Statistical analysis was performed using ordinary least squares linear regressions and the best fit model was displayed based on the maximum regression coefficient (The sections with gray shading as 95% prediction interval). The dotted line represents the non-significant relationship.

**Legends for supplementary tables S1 to S11**

**Table S1** Location of sampling points and edaphic information.

**Table S2** Primers used for functional genes sequencing and corresponding targeting sites.

**Table S3** Network topological structure properties for the bipartite networks.

**Table S4** Environmental and biotic variables used to explain bacterial beta diversity.

**Table S5** Formula for calculating composite variables for partial least squares path modeling (PLS-PM) of bacterial community in total sites (n=73).

**Table S6** Formula for calculating composite variables for partial least squares path modeling (PLS-PM) of bacterial community in low latitudes (n=38).

**Table S7** Formula for calculating composite variables for partial least squares path modeling (PLS-PM) of bacterial community in high latitudes (N=35).

**Table S8** Spearman rank correlation analysis showing the relationships of microbial (Bacteria, Protists and T4-like virus) richness and community structure with environmental attributes.

**Table S9** Spearman’s correlation of geographic distance, abiotic and biotic variables with bacterial community structure as determined by the Mantel test (9,999 permutations).

**Table S10** Pearson correlation analysis of the environmental factors and latitude.

**Table S11** ADONIS and ANOSIM analyses of the microbial composition based on Bray-Curtis distance in microcosms.

**Supplementary references**

[1] Schemske DW, Mittelbach GG, Cornell HV, Sobel JM, Roy K. Is there a latitudinal gradient in the importance of biotic interactions? Annu Rev Ecol Evol S. 2009; **40:** 245-269.

[2] Johnke J, Baron M, de Leeuw M, Kushmaro A, Jurkevitch E, Harms H, Chatzinotas A. A generalist protist predator enables coexistence in multitrophic predator-prey systems containing a phage and the bacterial predator. Front Ecol Evol. 2017; **5:** 124.

[3] Geisen S, Mitchell EAD, Adl S, Bonkowski M, Dunthorn M, Ekelund F et al. Soil protists: a fertile frontier in soil biology research. FEMS Microbiol Rev. 2018; **42:** 293-323.

[4] Liang XL, Wagner RE, Zhuang J, DeBruyn JM, Wilhelm SW, Liu F et al. Viral abundance and diversity vary with depth in a southeastern United States agricultural ultisol. Soil Biol Biochem. 2019; **137:** 107546.

[5] Li Y, Liu HY, Pan H, Zhu XY, Liu C, Zhang QC et al. T4-type viruses: Important impacts on shaping bacterial community along a chronosequence of 2000-year old paddy soils. Soil Biol Biochem. 2019; **128:** 89-99.

[6] Rosenberg K, Bertaux J, Krome K, Hartmann A, Scheu S, Bonkowski M. Soil amoebae rapidly change bacterial community composition in the rhizosphere of Arabidopsis thaliana. ISME J. 2009; **3:** 675-684.

[7] Wang YJ, Gong Q, Wu YY, Huang F, Ismayil A, Zhang DF et al. A calmodulin-binding transcription factor links calcium signaling to antiviral RNAi defense in plants. Cell Host Microbe. 2021; **29:** 1393.

[8] Lee SM, Kong HG, Song GC, Ryu CM. Disruption of Firmicutes and Actinobacteria abundance in tomato rhizosphere causes the incidence of bacterial wilt disease. ISME J. 2021; **15:** 330-347.

[9] Fine PVA. Ecological and evolutionary drivers of geographic variation in species diversity. Annu Rev Ecol Evol S. 2015; **46:** 369-392.

[10] Dynesius M, Jansson R. Evolutionary consequences of changes in species' geographical distributions driven by Milankovitch climate oscillations. P Natl Acad Sci USA. 2000; **97:** 9115-9120.

[11] Jansson R, Dynesius M. The fate of clades in a world of recurrent climatic change: Milankovitch oscillations and evolution. Annu Rev Ecol Syst. 2002; **33:** 741-777.

[12] Bahram M, Hildebrand F, Forslund SK, Anderson JL, Soudzilovskaia NA, Bodegom PM et al. Structure and function of the global topsoil microbiome. Nature. 2018; **560:** 233.

[13] Rose JM, Vora NM, Countway PD, Gast RJ, Caron DA. Effects of temperature on growth rate and gross growth efficiency of an Antarctic bacterivorous protist. ISME J. 2009; **3:** 252-260.

[14] Angly FE, Felts B, Breitbart M, Salamon P, Edwards RA, Carlson C et al. The marine viromes of four oceanic regions. Plos Biol. 2006; **4:** 2121-2131.

[15] Sinsabaugh RL, Lauber CL, Weintraub MN, Ahmed B, Allison SD, Crenshaw C et al. Stoichiometry of soil enzyme activity at global scale. Ecol Lett. 2008; **11:** 1252-1264.

[16] Noble RT, Fuhrman JA. Virus decay and its causes in coastal waters. Appl Environ Microb. 1997; **63:** 77-83.

[17] Bratbak G, Heldal M, Norland S, Thingstad TF. Viruses as partners in spring bloom microbial trophodynamics. Appl Environ Microb. 1990; **56:** 1400-1405.
